# Classification of *Bacillus* and *Brevibacillus* species using rapid analysis of lipids by mass spectrometry

**DOI:** 10.1007/s00216-016-9890-4

**Published:** 2016-09-07

**Authors:** Najla AlMasoud, Yun Xu, Drupad K. Trivedi, Simona Salivo, Tom Abban, Nicholas J. W. Rattray, Ewa Szula, Haitham AlRabiah, Ali Sayqal, Royston Goodacre

**Affiliations:** 1 School of Chemistry and Manchester Institute of Biotechnology, University of Manchester, 131 Princess Street, Manchester, M1 7DN UK; 2 Shimadzu, Kratos Analytical Ltd. Wharfside, Trafford Wharf Road, Manchester, M17 1GP UK; 3 Department of Pharmaceutical Chemistry, College of Pharmacy, King Saud University, P.O. Box 2457, Riyadh, 11451 Saudi Arabia

**Keywords:** MALDI-TOF-MS, LC-MS, Lipids, *Bacillus*, *Brevibacillus*, Classification, Extraction

## Abstract

*Bacillus* are aerobic spore-forming bacteria that are known to lead to specific diseases, such as anthrax and food poisoning. This study focuses on the characterization of these bacteria by the detection of lipids extracted from 33 well-characterized strains from the *Bacillus* and *Brevibacillus* genera, with the aim to discriminate between the different species. For the purpose of analysing the lipids extracted from these bacterial samples, two rapid physicochemical techniques were used: matrix-assisted laser desorption/ionisation time-of-flight mass spectrometry (MALDI-TOF-MS) and liquid chromatography in conjunction with mass spectrometry (LC-MS). The findings of this investigation confirmed that MALDI-TOF-MS could be used to identify different bacterial lipids and, in combination with appropriate chemometrics, allowed for the discrimination between these different bacterial species, which was supported by LC-MS. The average correct classification rates for the seven species of bacteria were 62.23 and 77.03 % based on MALDI-TOF-MS and LC-MS data, respectively. The Procrustes distance for the two datasets was 0.0699, indicating that the results from the two techniques were very similar. In addition, we also compared these bacterial lipid MALDI-TOF-MS profiles to protein profiles also collected by MALDI-TOF-MS on the same bacteria (Procrustes distance, 0.1006). The level of discrimination between lipids and proteins was equivalent, and this further indicated the potential of MALDI-TOF-MS analysis as a rapid, robust and reliable method for the classification of bacteria based on different bacterial chemical components.

**Electronic supplementary material:**

The online version of this article (doi:10.1007/s00216-016-9890-4) contains supplementary material, which is available to authorized users.

**Graphical abstract:**
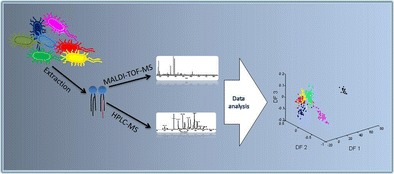
MALDI-MS has been successfully developed for the characterization of bacteria at the subspecies level using lipids and benchmarked against HPLC

## Introduction

Classification of bacteria has recently received increasing attention, most likely arising from public health concerns, environmental monitoring, food safety monitoring, taxonomic identification and differentiation of pathogenic species from non-pathogenic species, as well as for the identification of biological threat agents [1–3]. Bacteria can be classified using various physicochemical approaches based on different methods that rely either on analysis of: (1) protein from whole bacterial cells [4–9] or (2) extracts of different compounds, including (as in the current study) lipids [10–13]; each of these methods has its advantages and disadvantages.

Lipids are important components in bacterial cell membranes as they form lipid bilayers responsible for cell integrity [14, 15]. These cell components have various structures, and several factors can affect lipid synthesis such as culture media, temperature and physical dynamics during cell growth [16]. Complex lipids, just like fatty acids and proteins, can be used to identify and characterize bacteria [17, 18]. Interest in the analysis of lipid profiles from bacterial cells for taxonomic identification is increasing [11]. Not only do lipids play a structural role in the integrity of cell membranes, but they also contribute to other cellular processes such as metabolic and signalling pathways [19, 20].

Early studies that aimed to resolve lipid species traditionally used different chromatographic techniques such as thin layer chromatography [21]. This approach has disadvantages such as limited resolution and sensitivity which negatively affect many lipidomic applications [21]. Therefore, an armoury of techniques has been used to address many of these issues, which has led to the use of mass spectrometry technology, including direct infusion mass spectrometry [22] and liquid chromatography–mass spectrometry (LC-MS) [23], which have been extensively used to analyse lipid samples enabling the detection of different types of lipids. Matrix-assisted laser desorption/ionisation time-of-flight mass spectrometry (MALDI-TOF-MS) has also been used for lipidomic analysis to overcome the limitations seen with other traditional methods and to analyse samples containing complex mixtures of lipids, enabling classification and identification of bacteria [5, 11, 24]. The main advantages of MALDI-TOF-MS include: (1) it uses soft ionisation, causing minimal analyte degradation; (2) it offers the possibility to analyse a range of complex molecules in complex mixtures such as bacterial samples; (3) it requires minimal sample preparation; and (4) it yields mass spectra that contain specific chemical features and fingerprints that can be used to identify and characterize bacterial species [25, 26].

The aim of this study was to classify 33 strains of bacteria belonging to seven species—namely *Bacillus amyloliquefaciens*, *Bacillus cereus*, *Brevibacillus laterosporus*, *Bacillus licheniformis*, *Bacillus megaterium*, *Bacillus sphaericus* and *Bacillus subtilis*—based on the MALDI-TOF-MS of extracted bacterial lipids. The results of which were evaluated and validated using LC-MS to confirm the bacterial classification based on MALDI-TOF-MS analysis.

## Materials and methods

### Chemicals and solvents

Chemicals used were of a high purity grade and included the following: HPLC grade chloroform (Sigma-Aldrich, Dorset, UK), HPLC water (Sigma-Aldrich), HPLC grade methanol (Fisher Scientific Ltd., Loughborough, UK) and 99.99 % pure formic acid (VWR International, East Grinstead, UK). Two different matrices were used in this study: 6-aza-2-thiothymine (ATT) and 2,5-dihydroxybenzoic acid (DHB; both from Sigma-Aldrich).

### Microorganisms

Seven bacterial species (i.e. *B. amyloliquefaciens*, *B. cereus*, *Br. laterosporus*, *B. licheniformis*, *B. megaterium*, *B. sphaericus* and *B. subtilis*) were used in this study. Table 1 gives details of these 33 strains from the *Bacillus* and *Brevibacillus* genera; these were used previously in MALDI-TOF-MS analysis of bacterial proteins [4].

**Table 1:** The 33 *Bacillus* and *Brevibacillus* species and strains used in this study

Sample no.	Species	Strain no.	Key colour in figures
1	*B. amyloliquefaciens*	B0177^T^	Red
2		B0168	
3		B0175	
4		B0251	
5		B0620	
6	*B. cereus*	B0002^T^	Green
7		B0550	
8		B0702*	
9		B0712	
10		B0851	
11	*B. licheniformis*	B0252^T^	Blue
12		B0242	
13		B0755	
14		B1081	
15		B1379	
16	*B. megaterium*	B0056	Cyan
17		B0057	
18		B0076	
19		B0621	
20	*B. sphaericus*	7134^T^	Pink
21		B0408	
22		B0219	
23		B0769	
24		B1147	
25	*B. subtilis*	B0014^T^	Yellow
26		B0044	
27		B0098	
28		B0099*	
29		B0410	
30		B0501	
31		B1382	
32	*Br. laterosporus*	B0043*	Black
33		B0262	

* indicates strains used for preliminary optimisation experiments for time points in LC-MS; ^T^ indicates the type of strain

### Bacterial cultivation

Using sterile plastic loops bacterial strains were cultivated three times for 24 h at 37 °C on nutrient agar (NA) to generate axenic colonies and to maintain a stable phenotype. NA contained beef extract 3 g/L, peptone 5 g/L, NaCl 8 g/L and Agar no. 2 at 12 g/L from Lab-M (Bury, UK) and was prepared following the manufacturer’s instructions (28 g in 1 L of deionised water) and subsequently autoclaved (121 °C and 15 psi for 15 min) before Petri dishes were prepared.

### Optimisation of collection time points for three different species of *Bacillus* and *Brevibacillus* for LC-MS

Three different species were used at the beginning of this work to choose the optimal collection time points; these species were *B. cereus* B0702, *B. subtilis* B0099 and *Br. laterosporus* B0043. An axenic colony was collected from each culture and inoculated in 600 mL of nutrient broth (prepared according to the manufacturer’s instructions; Oxoid Ltd., Basingstoke, UK) in 2 L flasks and then incubated for 24 h at 37 °C at 200 rpm. Optical density (OD) measurements at 600 nm were collected at six different time points (4, 6, 8, 10, 14 and 18 h) using a Biomate 5 spectrophotometer (Thermo-Fisher Ltd., Hemel Hempstead, UK). For each species, three biological replicates were prepared in the same way.

### Quenching

Samples were collected at the six different time points (4, 6, 8, 10, 14 and 18 h). From each culture, 15 mL was quenched using 30 mL of 60 % cold methanol (−48 °C, chilled on dry ice) and mixed rapidly. This was followed by centrifugation of the quenched culture for 10 min at 4800 × *g* at −8 °C [27]. The supernatant was removed quickly and then the rest was centrifuged again for 2 min and the remaining supernatant removed, leaving the pellet containing the bacterial cells in the centrifuge tube. The pellets were stored at −80 °C until lipid extraction was performed [13]. Figure S1A in the Electronic supplementary material (ESM) illustrates this process.

### Lipid extraction

Bacterial pellets were mixed with 2 mL HPLC grade chloroform/methanol (2:1) pre-chilled at −20 °C. The samples were mixed using a laboratory shaker for 15 min, and 1 mL of cold HPLC water was then added to the mixtures. This was followed by centrifugation at 4800 × *g* for 3 min at −8 °C [27]. A biphasic system was generated, with the bottom chloroform-based layer containing most of the lipids. The lipid layers were transferred to fresh 2 mL microcentrifuge tubes [10]. The samples were left to evaporate on a hot plate at 40 °C to complete dryness prior to storage at −80 °C (ESM Fig. S1B). These samples were reconstituted in 80:20 methanol/water (*v/v*) at 100 μL per 0.1 OD_600_ and then analysed using LC-MS.

### Collections of *Bacillus* and *Brevibacillus* strains for LC-MS and MALDI-TOF-MS analysis

In total, 33 strains were collected for LC-MS and MALDI-TOF-MS after 10 h of culturing at 37 °C and 200 rpm. Five biological replicates were collected for each strain.

### Preparing extracted samples for MALDI-TOF-MS

For MALDI analysis of the extracted lipids, the samples were reconstituted in 80:20 methanol/HPLC water (*v/v*). Of the DHB, 10 mg was dissolved in 900 μL ethanol and 100 μL sterile deionised water, and 10 mg of ATT was dissolved in 500 μL acetonitrile and 500 μL of sterile deionised water. Ten microlitres of the extracted lipid samples was mixed with 10 μL of either matrix, and then 2 μL of the matrix/samples mixture was applied to a MALDI stainless steel plate and allowed to dry at room temperature (approx. 22 °C).

### MALDI-TOF-MS analysis

Samples were analysed in batches using an AXIMA-Confidence mass spectrometer (Shimadzu Biotech, Manchester, UK) equipped with a nitrogen pulsed UV laser (337 nm) [4] set at 100 mV; each profile was produced using 20 laser shots, and 78 profiles were collected using a circular raster pattern. The instrument was operated in positive ionisation mode using the reflectron TOF over the mass-to-charge ratio (*m*/*z*) range 100–1600. Each biological sample was analysed in four technical replicates. A single biological replicate of each of the 33 bacterial strains was analysed each day. Before sample analysis, the MALDI instrument was calibrated with polyethylene glycol using the following *m*/*z* values: 613.7, 657.75, 710.80, 746.86, 789.91, 833.96, 878.02, 922.07, 966.12, 1010.18, 1054.23, 1098.28, 1142.34, 1186.39, 1230.44, 1274.50, 1318.55 and 1362.60.

### Sample preparation of MALDI-TOF/TOF

Sample preparation was carried out as follows. Samples were reconstituted in 1:1 chloroform/methanol (*v*/*v*). DHB was used as matrix and was prepared in methanol (10 mg/mL) containing 10 mM NaCl. A sample droplet (0.35 μL) was placed onto a MALDI target spot, followed by an equal amount of matrix solution.

### MALDI-TOF/TOF analysis

The samples were analysed on a MALDI 7090 mass spectrometer (Shimadzu Kratos, Manchester, UK) with a solid-state UV laser (355 nm) operating at a 2 kHz acquisition repetition rate. The instrument was operated at an acceleration voltage of 20 keV, and a pulsed extraction function to improve mass resolution was carefully applied. The low mass rejection and the focus mass were set to 300 and 800 Da, respectively. The instrument was operated in the reflectron mode. To enhance the signal-to-noise ratio, 100 single shots were averaged for each mass spectrum. Laser intensity was adjusted for each experiment to obtain the best signal-to-noise ratio and to maximize the number and intensity of structural fragments. Positive mode spectra of all analytes were recorded. Helium gas was used for high-energy CID (20 keV) MS/MS experiments. All mass spectrometric data were acquired and analysed using the MALDI Solution software (Shimadzu Kratos).

### LC-MS analysis

An Accela UHPLC system (Thermo-Fisher Ltd.) coupled to an electrospray LTQ-Orbitrap XL hybrid mass spectrometry system (Thermo-Fisher, Bremen, Germany) was used to analyse the samples. Samples were reconstituted in 80:20 methanol/HPLC water based on 100 μL per OD_600_ of 0.1. The mixture was vortexed and centrifuged at 11,500 × *g* for 30 s. Quality control (QC) samples were prepared by mixing an equal volume of each extracted sample and vortexing the mixture thoroughly. The mixtures were then transferred to 100 μL analytical vials [28]. All samples were run in positive electrospray ionisation (ESI) mode since LC-MS was used to confirm the results obtained from MALDI-TOF-MS, which was also operated in the positive ionisation mode.

First, three biological replicates were analysed over 5 days and the remaining two biological replicates analysed over a further 3-day period to account for the large number of samples. Briefly, 10 μL of extracted sample was injected onto a Hypersil GOLD UHPLC C_18_ analytical column (length, 100 mm; diameter, 2.1 mm; particle size, 1.9 μm; Thermo-Fisher Ltd.). The flow rate used for UHPLC was 400 μL/min. The two solvents used for LC were water with 0.1 % formic acid (solvent A) and methanol with 0.1 % formic acid (solvent B). The following settings were used for chromatographic separation in positive ionisation mode: 100 % A held for 1 min, 0–100 % B over 11 min, 100 % B held for 8 min, returning to 100 % A over 2 min (total run time, 22 min). The column was conditioned prior to analysis by running 50:50 water/methanol gradient in isocratic conditions for 3 h at 50 °C followed by 30 min of initial gradient conditions. All the samples were analysed with column temperature at 50 °C. Xcalibur software (Thermo-Fisher Ltd.) was used to operate the Thermo LTQ-Orbitrap XL MS system using the same method described by Wedge et al. [23]. The LTQ-Orbitrap MS was calibrated according to the manufacturer’s instructions. Orbitrap data were obtained at a resolution of 30,000 (FWHM defined at *m*/*z* 400).

### Orbitrap MS^*n*^ analysis parameters

Direct infusion of samples was carried out onto a LTQ-Orbitrap XL hybrid mass spectrometry system (Thermo-Fisher) in order to conduct MS^*n*^ experiments. Samples were injected at a constant flow of 10 μL/min into ESI probe. A full scan of the sample was followed by trapping the ion of interest in an ion trap for 30 ms and collision-induced fragmentation was carried out with varied CID levels (between 35 and 200 arbitrary units). This was repeated until no more fragmentation could be carried out for the precursor ion in each cycle.

The batch programme involved the use 20 injections of QC samples for each individual analytical block. These were used for column conditioning. The analysis batch then followed, where five injections of extracted samples were followed by a QC injection. These steps were repeated until all the samples were analysed and the run was concluded by performing three QC injections.

### Processing raw data and generating UHPLC-MS profiles

Xcalibur software’s file conversion option was used to convert the raw data profiles obtained using UHPLC-MS into a NetCDF format [29]. A free package for R available from http://masspec.scripps.edu/xcms/xcms.php was used to deconvolute the peaks using in-house deconvolution parameters fit for the high-resolution mass spectrometric data collected. Once the peaks were deconvoluted, a Microsoft Excel sheet (XY) matrix was produced containing spectral features including: retention time and *m*/*z* ratios. The total numbers of mass spectral features from the LC-MS data were 2618. After deconvolution, lipid identification was carried out using Taverna Workbench version 2.4 [23].

### Statistical analysis of data

#### Analysis of MALDI-TOF-MS data

All data pre-processing and data analysis were carried out using MATLAB 2012a (MathWorks, Natick, MA, USA). MALDI-TOF-MS spectra were subjected to the following pre-processing steps: (1) baseline correction using asymmetric least squares [30] of the raw MS data and (2) normalization carried out by dividing the baseline-corrected spectrum with the square root of the sum of squares of the spectrum. Multivariate analysis included principal components discriminant function analysis (PC-DFA) [31] and partial least squares for discriminant analysis (PLS-DA) [32, 33]. PLS-DA with 1000 bootstraps was performed. In this process, the data were split into two different sets: a training set and a test set using bootstrap resampling based on biological replicates, as described before [4, 34].

The LIPID MAPS online database was used to identify the lipid peaks based on accurate mass information from MALDI-TOF-MS analysis (http://www.lipidmaps.org/).

#### Analysis of LC-MS data

PC-DFA and PLS-DA were also performed on LC-MS data, and PLS-DA modelling was also validated using bootstrap resampling. In order to identify the most significant lipids features, PC-DFA and PLS-DA loading plots were used.

#### Comparison of two analytical techniques

MALDI-TOF-MS and LC-MS results were then compared using the Procrustean test [35]. The test was based on Procrustes analysis, which is an effective approach for assessing the similarities and differences between different ordination spaces from cluster analyses and has been used previously for the assessment of different analytical techniques [13]. In Procrustes analysis, the similarity between two sets of multivariate datasets, i.e. two matrices with the same number of rows, was measured in terms of the Procrustes distance, which ranges between 0 and 1, where 0 indicates a perfect match and 1 indicates nothing in common. The Procrustes test on the two datasets was based on such Procrustes distance. Given two data matrices, a Procrustes distance was calculated (named observed Procrustes distance), and this distance was then compared against a null distribution generated by *n* permutations. In each permutation, the order of the rows in one matrix (e.g. MALDI-TOF-MS lipid) was randomly permuted whilst that of the other (e.g. LC-MS lipid) remained the same; a Procrustes distance was then calculated. A total number of *n* Procrustes distances were calculated from *n* different random permutations and formed the null distribution. An empirical *p* value was then calculated by counting the cases where the Procrustes distance from the null distribution was lower than the observed Procrustes distance. In this study, we compared three datasets, i.e. MALDI-TOF-MS lipids, MALDI-TOF-MS protein and LC-MS lipids, using Procrustes tests. For each test, 1000 permutations were performed and the observed Procrustes distance and the associated *p* values were reported.

## Results and discussion

Traditional phenotypic methods such as biochemical tests [7] are used routinely to discriminate between different microorganisms. These methods, however, are not always reliable and are generally laborious, time-consuming and provide limited information compared to modern analytical techniques [13, 21, 36]. For the purpose of this lipidomic study, two complementary analytical techniques were used to analyse lipids extracted from 33 *Bacillus* and *Brevibacillus* strains—MALDI-TOF-MS and LC-MS. The findings of this work show that the use of MALDI-TOF-MS to classify bacteria based on lipid extracts is promising and can be a useful analytical tool for research carried out in the lipidomics field.

At the beginning of this work, three different species (*B. cereus* B0702, *B. subtilis* B0099 and *Br. laterosporus* B0043) were analysed using LC-MS to determine the optimal time point for collecting bacterial samples based on the quality of separation determined using LC-MS data. Our observations show that samples collected after 10 h of cell culture (ESM Fig. S2A, B) generated better separation for the three species due to there being a sufficient amount of biomass that is needed for lipid extraction, which was evident from the OD (data not shown).

### MALDI-TOF-MS lipid profiles

Recently, we optimised the experimental conditions for the detection of lipid mixtures using MALDI-TOF-MS analysis and fractional factorial design [37]. Our observations suggested that ATT and DHB were the most compatible matrices with lipid mixtures when studies were carried out in the positive ion mode. We note here that some preliminary experiments in negative ion mode with DHB were conducted, but that the resulting spectra were not very information-rich and certainly did not contain as many mass ion features as the data from the same extract collected in positive ion mode (data not shown). Initially, as routine practice in our laboratory when conducting MALDI-TOF-MS experiments, pilot tests were performed before analysing samples. In this case, two different species, *B. cereus* and *B. subtilis*, were used and lipid extracts were analysed with MALDI-TOF-MS using the two matrices ATT [38, 39] and DHB [40, 41] as these were found to be the most compatible matrices with the lipid mixture. Figure S3 in the ESM shows the principal component analysis (PCA) scores plot of *B. cereus* and *B. subtilis* using these matrices, and the results suggested that DHB provided better separation between the bacteria based on the total explained variance (TEV) values generated in PC1 dimension in the PCA plots, which were higher at around 84 % compared to 54 % achieved with ATT. Previous studies showed that DHB is a compatible matrix with lipid analysis as DHB matrix peaks do not interfere with the interpretation of spectral data [42, 43]. A relatively good separation between bacterial samples was still generated using ATT; however, due to the huge number of samples, only the better performing matrix (DHB) was used in order to generate more reliable data for all of the 33 *Bacillus* and *Brevibacillus* strains.

We appreciate that performing lipid extracts on bacteria can increase sampling time; therefore, before analysis of all bacteria, we also carried out direct MALDI-TOF-MS analysis for lipids from intact bacterial cells. However, the spectra we generated from the analysis of intact bacteria were very poor in terms of reproducibility and spectral quality, that is to say the number of mass ions detected (data not shown). This intact analysis may be compromised due to the interference from other cellular components, such as proteins being preferentially ionised, and thus may lead to ion suppression of lipids; even though the spectra are collected in the low mass range (300 and 800 *m*/*z*), higher-molecular-weight species will compete for ionisation. Therefore, the extra steps required for lipid extraction and sample preparation are a necessary inconvenience to acquire higher quality data.

**Fig. 1:**
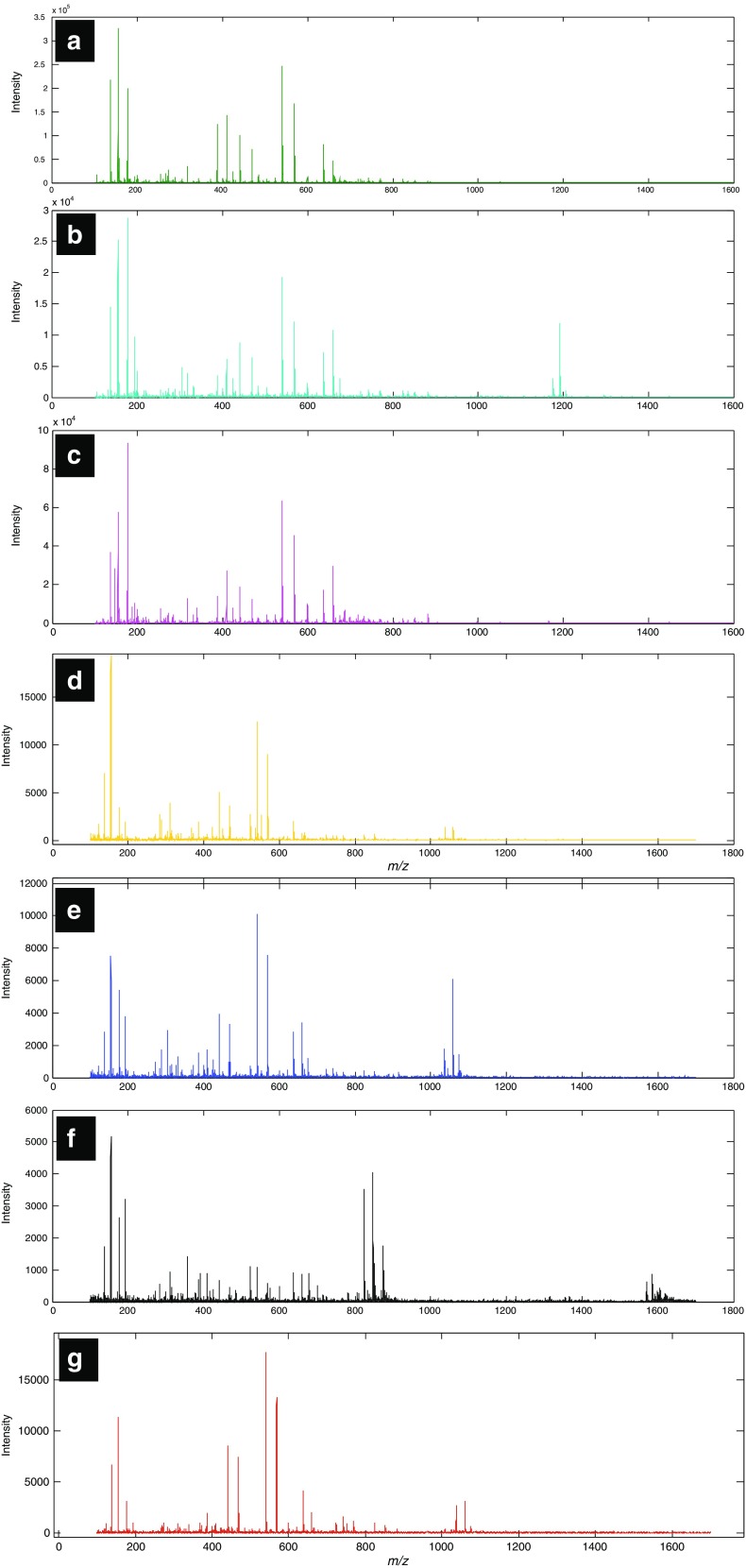
Typical MALDI-TOF-MS after pre-processing of lipids extracted from seven species: *B. cereus* B0002 (**a**); *B. megaterium* B0056 (**b**); *B. sphaericus* B0769 (**c**); *B. subtilis* B1382 (**d**); *B. licheniformis* B1379 (**e**); *Br. laterosporus* B0034 (**f**); and *B. amyloliquefaciens* B0177 (**g**)

Lipids were extracted from *Bacillus* and *Brevibacillus* using chloroform/methanol (2:1) since this method was used previously with successful outcomes [10, 12, 44]. The MALDI-TOF-MS spectra of lipids extracted from all seven species—*B. amyloliquefaciens* B0177, *B. cereus* B0002, *Br. laterosporus* B0034, *B. licheniformis* B1379, *B. megaterium* B0056, *B. sphaericus* B0769 and *B. subtilis* B1382—are shown in Fig. 1. In general, MALDI-TOF-MS generated high-quality data due to the high signal-to-noise ratios over the *m*/*z* range of acquisition.

At first glance, the MALDI-TOF-MS spectra for the seven species from *Bacillus* and *Brevibacillus* appeared to have different patterns in the *m*/*z* range 200–1600 (Fig. 1). Some parts of the spectra were amplified to show peaks that cannot be visualized due to low intensities in comparison to the more dominant peaks. These spectra are rich in information, and lipids were detected across a broad range, mainly below *m*/*z* 1600. Some of the peaks remained the same for the seven species, for example lipids at *m*/*z* values of 568, 637, 659 and 851. On the other hand, other parts of the spectra are unique to each species, such as the region between *m*/*z* 1500 and 1600 in *Br. laterosporus*. Visual inspection of the MALDI-TOF-MS spectra revealed features that can be used to discriminate between some of the species. *Br. laterosporus* was characterized by significantly different spectra compared to the other species, most likely due to the expected differences between bacterial genera [45]. It is important to note that the biomass concentration was the same for the seven species analysed in this study. However, the signal-to-noise ratios seemed to be different from one spectrum to another; this is possibly due to the ionisation efficiency of analytes under MALDI-TOF-MS analysis and can possibly be assessed using different matrices.

Figure 1A–G shows that during the growth of bacterial strains in nutrient broth, they produced lipids represented by the detection of various peaks on different spectra. These peaks, which were readily detectable by a simple MALDI-TOF-MS analysis, may represent significant lipids that can be used as a fingerprint for each type of bacteria. The LIPID MAPS database (http://www.lipidmaps.org/) was used to assign the most abundant lipid peaks; the probable assignments for the seven species are listed in Table 2. Table 2 also shows that sodium and potassium adducts can be seen in the MALDI mass spectra owing to the nature of the biological samples, which are rich in these cations. Lipids detected in these species are a broad set of naturally occurring molecules. Several studies have confirmed that phosphatidylethanolamine (PE) and phosphatidylglycerol (PG) are the most abundant phospholipids in bacteria such as *Bacillus* spp. [44, 46, 47] and *Escherichia coli* [44]. *Bacillus* has also been reported to produce other categories of lipids such as digalactosyldiacylglycerol (DGDG) [11, 44], phosphatidylcholine (PC) [11, 44, 48] and fatty acids [49]. These significant lipid features were subjected to MS/MS analysis and MS^*n*^ analysis on MALDI-TOF/TOF, as well as Orbitrap MS respectively in order to obtain structural information to validate our putative assignments. It was noted that not all lipid features that were in significant abundance required MS^*n*^ analysis. Table 2 includes the lipid features present in the seven species classified in this study. Identification of lipids was based on accurate mass match on LIPID MAPS, followed by verification of their presence reported in the literature and further confirmation by MS^*n*^ analysis.

**Table 2:**
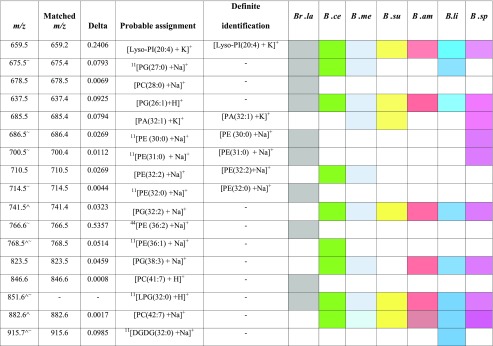
List of probable and definite identification of the seven *Bacillus* species using MS*n* fragmentation results

If a peak was detected for a particular lipid, this is illustrated with a colour matching the different species

*B.amy B. amyloliquefaciens*, *B.cer B. cereus*, *Br.lat Br. laterosporus*, *B.lic B. licheniformis*, *B.meg B. megaterium*, *B.sph B. sphaericus*, *B.sub B. subtilis*, *PC* phosphatidylcholine, *PE* phosphatidylethanolamine, *PA* phosphatidic acid, *PG* phosphatidylglycerol, *LPI* l-alpha-lysophosphatidylinositol, *LPG* lyso-phosphatidylglycerol, *DGDG* digalactosyldiacylglycerol

Caret (^) indicates *m/z* of lipid-like features that were not in high enough quantities to be able to successfully fragment into product ions

Tilde (∼) indicates *m/z* of features that have been putatively assigned an identification based on previous reports in the literature (ref 11 or 44; in column 4)

With regard to the structural identification carried out by means of tandem MS, the high-energy CID MS/MS (MALDI-TOF-MS) and MS^*n*^ (Orbitrap) spectra exhibited the characteristic fragmentation of the polar head group of the phospholipids. Specifically, ions equivalent to [M−43]^+^, [M−141]^+^ and [M−163]^+^ [50, 51], corresponding to the loss of ethanolamine, ethanolamine phosphate and sodiated ethanolamine phosphate, respectively, were consistently observed in the tandem MS spectra of PE lipids. In the MS/MS and MS^*n*^ spectra of PA lipids (one single species has been found), the loss of phosphate ([M–98]^+^) and potassium phosphate group ([M−136]^+^) has been observed accordingly.

Out of 17 lipids, six lipids were assigned definite identification based on their fragmentation pattern, whereas five lipids were observed in insufficient quantities to be able to perform fragmentation. There were six lipids that were only identified based on their accurate mass as their fragmentation pattern did not follow a lipid-like fragmentation. Putatively identified lipids were assigned identification based on either their match on LIPID MAPS or previous reports of successful fragmentation by other authors using various fragmentation techniques.

Figure 1A shows a zoomed-in area that contains mass peaks between around *m*/*z* 600 and 800 in the *B. cereus* spectrum, representing lipids consisting of different numbers of carbon, from different categories such as PE, PG and PC. The spectrum generated from *B. megaterium* (Fig. 1B) seemed to be similar to *B. cereus* based on the existence of PE, PG and PC, whilst *B. megaterium* produced a visibly unique peak at around 1206 *m*/*z*. Figure 1D shows the mass spectrum of *B. subtilis*, where fewer peaks were detected compared to *B. cereus* and *B. megaterium*. Notably, the spectrum in Fig. 1G, which represents *Br. laterosporus*, is largely dominated by peaks at *m*/*z* 1224, 1315, 1335, 1367, 1570 and 1584, a series of peaks that can be used to identify this species; the fact that this species is different is perhaps not surprising as these bacteria are from different genera.

**Fig. 2:**
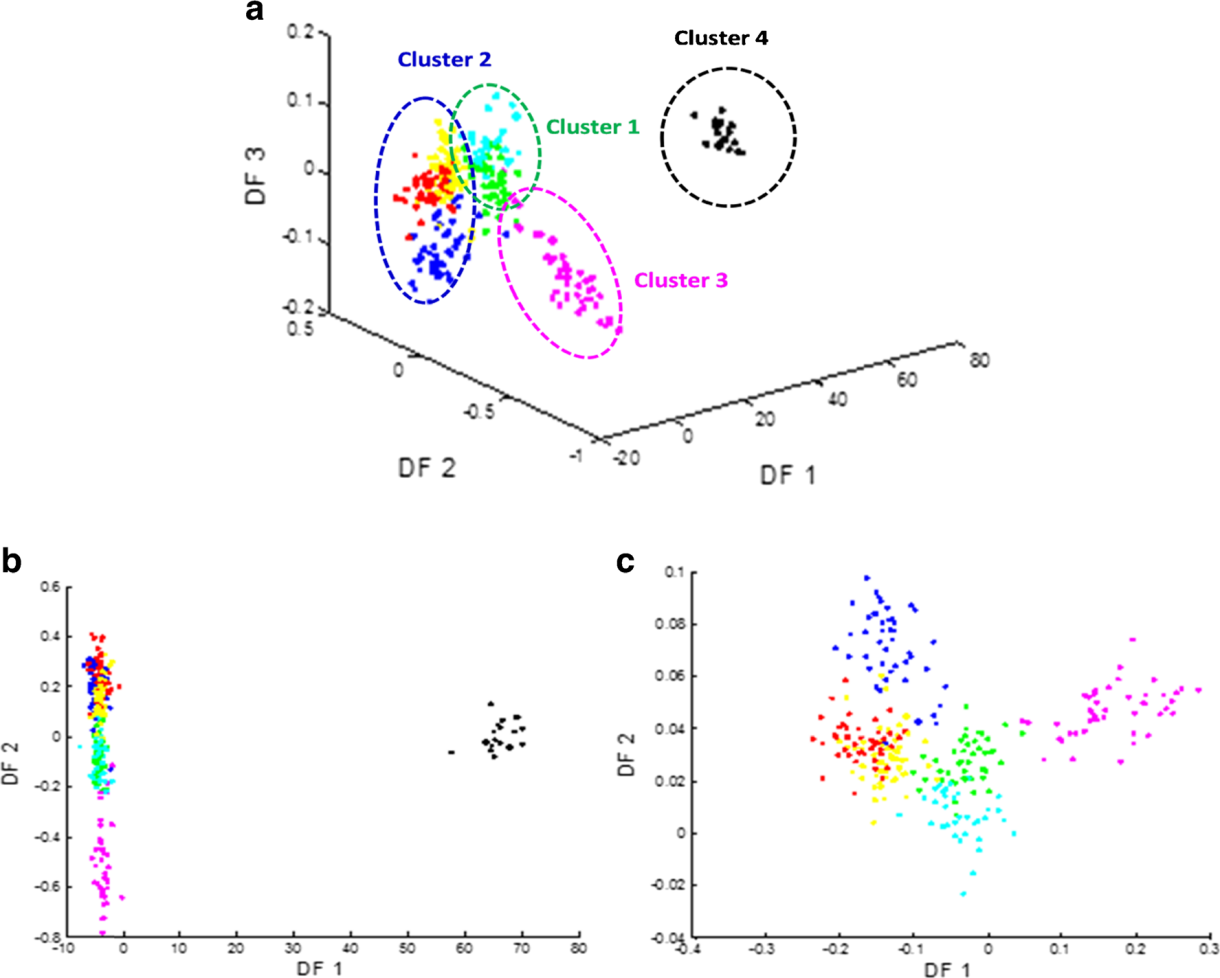
DFA scores plots after pre-processing MALDI-TOF-MS data. Different DFA plots were generated for seven species, including: DF1 vs. DF2 vs. DF3 (**a**); DF1 vs. DF2 (**b**); and DF1 vs. DF2 (**c**) of six species, with *Br. laterosporus* removed. *Different colours* represent different species. Table 1 details the annotations

However, visual inspection is laborious and unreliable; consequently, advanced chemometric methods were required to extract more information from the MS data in a reproducible, objective and automated manner. We have previously shown that after the optimisation of MALDI-TOF-MS in combination with advanced chemometrics, this analytical technique can become a robust and rapid tool that enables the classification of a large number of *Bacillus* and *Brevibacillus* bacterial strains based on their proteins [4]. Multivariate analysis has been proven vital for extracting information when analysing samples using different analytical techniques, such as pyrolysis mass spectrometry, Fourier transform infrared spectroscopy and Raman spectroscopy, to discriminate between bacterial samples [52–54]. There are different statistical methods that can be used to assess the information generated from the MALDI-TOF-MS spectra, enabling discrimination between the seven species. One such method is PC-DFA. In Fig. 2A, a three-dimensional DFA scores plot shows four major clusters detected based on the data: (1) *B. megaterium* and *B. cereus*; (2) *B. subtilis*, *B. amyloliquefaciens* and *B. licheniformis*; (3) *B. sphaericus*; and (4) *Br. laterosporus*. Figure 2B shows that *Br. laterosporus* is well separated in the first DF and is therefore completely chemically different from the other six *Bacillus* species, which confirms the differences seen in the MALDI-TOF-MS spectra. These large lipid differences in all the *Br. laterosporus* species dominated both plots, and therefore another PC-DFA plot was generated for *Bacillus* species only. This resulted in more separation between the six *Bacillus* species (Fig. 2C). Most notably, *B. licheniformis* could be separated from *B. subtilis* and *B. amyloliquefaciens* since *B. amyloliquefaciens* was shown to be similar to *B. subtilis*, which is expected as these two species are phylogenetically very closely related [55].

These *Bacillus* species were examined previously using different types of techniques, such as the analytical profile index (API) [45] and genotyping using 16S rDNA sequencing [52]. API test is used for bacterial classification based on miniaturized biochemical tests. Using API tests, four main groups were observed, including: group I with only *B. cereus*; group II containing only *Br. laterosporus*; group III containing only *B. sphaericus*; and a large group IV consisting of *B. subtilis*, *B. licheniformis*, *B. amyloliquefaciens* and *B. megaterium*. In contrast, phylogenetic analysis using 16S rDNA sequencing detected five different clusters: (1) *B. sphaericus*; (2) *Br. laterosporus*; (3) *B. subtilis*, *B. licheniformis*, and *B. amyloliquefaciens*; (4) *B. megaterium*; and (5) *B. cereus*.

The next stage in the present study was to assess whether the MALDI lipid profiles contained enough information for the identification of all the different bacteria. Therefore, automated classification prediction accuracies for the seven species and 33 strains were calculated based on the MALDI-TOF-MS data using multiple PLS-DA models. Table 3 and ESM Table S1 summarize the classification of *Bacillus* and *Brevibacillus* bacteria at the species level (i.e. seven classes) and at the strain levels (i.e. 33 classes), respectively. The average correct classification rate (CCR) for the seven species was 62.23 %, whereas the CCR for the 33 strains was 15.67 %. Interestingly, prediction of *Br. laterosporus* based on MALDI-TOF-MS data was more accurate compared to the other species. Moreover, if *B. amyloliquefaciens*, *B. subtilis* and *B. licheniformis* are considered as one class, as these species have the same phylogenetic origin [40], the prediction accuracies for the three species increase from 60.26, 67.76 and 59.60 % to 92.28, 91.61 and 87.45 %, respectively. Heat map plots from confusion matrices were generated using the PLS models from the seven species and 33 strains (ESM Fig. S4A, B, respectively). In these figures, warm colours (e.g. red) are indicative of species or strains of high percentage class membership assignments using MALDI-TOF-MS data, whilst cold colours (e.g. blue) represent low percentage class membership assignment. It can be seen that the colours on diagonal ‘tiles’ were generally much warmer than off-diagonal ‘tiles’, indicating high agreement between the predicted and known classes.

**Table 3:** Prediction accuracy of seven species from *Bacillus* using PLS-DA based on MALDI-TOF-MS data

	*B.amy*	*B.cer*	*B.lic*	*B.meg*	*B.sph*	*B.sub*	*Br.lat*
*B.amy*	60.26	5.85	5.80	1.25	0.50	26.22	0.01
*B.cer*	0.23	63.36	2.45	18.02	6.30	9.55	0.00
*B.lic*	10.51	8.52	59.60	2.36	1.58	17.34	0.00
*B.meg*	2.84	36.54	0.87	43.40	0.95	15.31	0.00
*B.sph*	0.02	19.17	0.16	0.23	80.11	0.21	0.00
*B.sub*	12.50	9.63	3.56	6.35	0.10	67.76	0.00
*Br.lat*	2.26	3.15	1.33	2.60	1.60	5.30	83.66

*B.am B. amyloliquefaciens*, *B.ce B. cereus*, *Br.la Br. laterosporus*, *B.li B. licheniformis*, *B.me B. megaterium*, *B.sp B. sphaericus*, *B.su B. subtilis*

The same bacterial species were previously classified based on MALDI-TOF-MS analysis of proteins from intact bacterial cells [4]. The overall classification based on protein analysis was highly similar to that based on the lipid analysis reported here. However, the quality of classification carried out based on protein analysis from intact cells was superior, with CCRs of over 80 % at the species level (average CCR of 89 %). This may be explained by the better quality of spectra obtained for proteins using MALDI-TOF-MS or the inherent differences in gene products between bacteria compared to those of metabolites, such as lipids. The case of misclassification of *B. megaterium* with *B. cereus* based on lipid profiles is interesting as these two species were very distinctly classified using protein profiles (CCRs of 91 and 83 %, respectively), indicating that the protein profiles were different whereas the lipid profiles were similar.

### Interpretation of LC-MS lipid profiles

Although MALDI-TOF-MS is a robust and rapid analytical technique, interference of matrix peaks with low-molecular-weight analyte peaks, especially those of lipids below 300 *m*/*z*, and its inability to discriminate between isobaric peaks (which have the same *m*/*z*) present a potential limitation to this chemotaxonomic technique. Therefore, LC-MS analysis was carried out on the same samples to complement and confirm the classification of bacteria based on MALDI-TOF-MS analysis. Although the mass accuracy (<10 ppm) of TOF analysers is high (∼15,000 full width at half maximum (FWHM) in reflectron mode), it is recognised that Orbitrap mass analysers have higher mass accuracy (sub-parts per million) and resolution (>100,000 FWHM), allowing the identification of lipids to be more accurate and robust [56]. The high mass accuracy and resolution of the Orbitrap combined with the resolution of analytes by HPLC can reduce the observed interference between the different lipid species and other components of the samples. These factors, considered together, are expected to lead to better classification and identification by LC-MS.

The LC-MS findings suggest that *Bacillus* species produced many different lipid categories, such as: phosphatidylcholine (PC), phosphatidylethanolamine (PE), diradylglycerolipid, glycerophosphoglycerol (PG), phosphatidic acids (PA), glycerophosphoinositol and ceramide. Methyl-branched fatty acids were observed in the lipid profiles of *Bacillus* species; these include dimethyl tetradecanoic acid (C15), methyl hexadecanoic acid (C17) and menaquinones, in line with previous reports [49, 57]. A summary of these putative lipid categories is shown in ESM Table S3. ESM Table S3 shows that the main lipids detected in LC-MS were most likely PE and PC, in addition to a small number of PA.

**Fig. 3:**
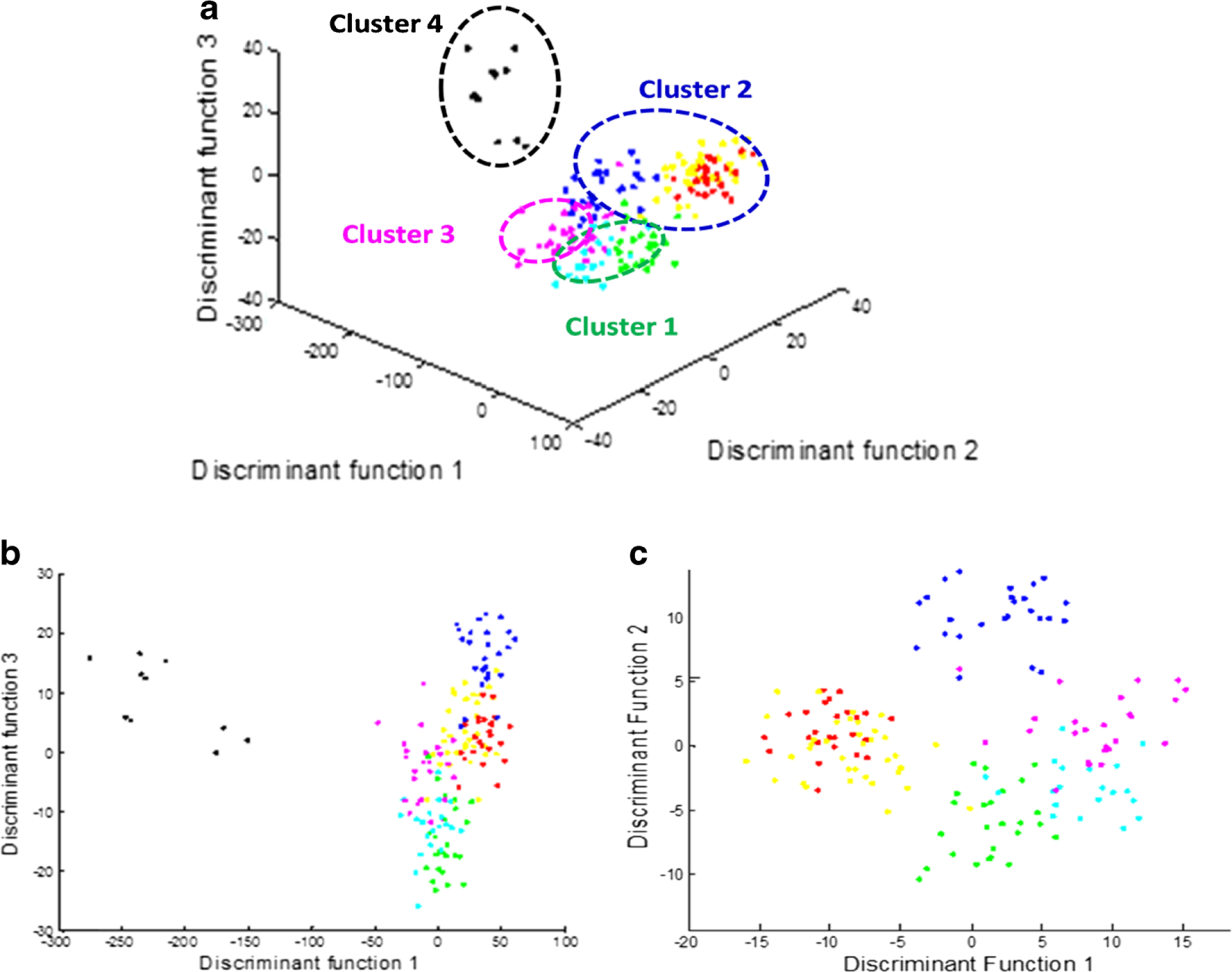
DFA scores plots after pre-processing the LC-MS data. Different DFA plots were generated for seven species, including: DF1 vs. DF2 vs. DF3 (**a**); DF1 vs. DF3 (**b**); and DF1 vs. DF2 (**c**) for the six species (again, *Br. laterosporus* was not included). *Different colours* represent different species. Table 1 shows the annotations

In order to compare classifications based on LC-MS data with those generated from MALDI-TOF-MS, PC-DFA was also applied to LC-MS data. Figure 3A shows a DFA scores plot of the 33 strains in three dimensions. It can be noted that four main clusters were detected: (1) *B. megaterium* and *B. cereus*; (2) *B. subtilis*, *B. amyloliquefaciens* and *B. licheniformis*; (3) *B. sphaericus*; and (4) *Br. laterosporus*. These observations were in agreement with MALDI-TOF-MS analysis based on these bacterial lipids (Fig. 2A). Moreover, this observation is similar to the previous work that we carried out based on whole cell analysis of proteins using MALDI-TOF-MS [4], Raman spectroscopy [3] and direct infusion ESI-MS [58]. Figure 3B shows that *Br. laterosporus* is again significantly different from the other strains when DF2 vs. DF3 and DF1 vs. DF3 are plotted. Therefore, *Br. laterosporus* was again excluded from data analysis, and this resulted in the separation of *B. licheniformis* from *B. subtilis* and *B. amyloliquefaciens* (Fig. 3C).

In order to achieve bacterial classification from these LC-MS lipid profiles, data analysis was carried out using a PLS-DA model for the seven species (i.e. seven classes) and 33 strains (i.e. 33 classes). Table 4 and ESM Table S2 show the prediction accuracies for the seven species and 33 strains, respectively. Table 4 shows that qualitative information based on lipids is appropriate for accurate classification of bacteria. This model provided average CCRs of 77.03 and 15.20 % for the seven species and 33 strains, respectively. Looking back at Table 3, which was generated from MALDI-TOF-MS data using the PLS-DA model, it can be observed that the results from these two analytical techniques overlapped and most of the species reflected higher predication accuracies based on LC-MS data due to the high sensitivity of LC-MS compared to MALDI-TOF-MS.

**Table 4:** Prediction accuracy of seven species from *Bacillus* using PLS-DA based on the LC-MS data

	*B.amy*	*B.cer*	*B.lic*	*B.meg*	*B.sph*	*B.sub*	*Br.lat*
*B.amy*	93.85	1.09	0.18	0.16	0.10	4.62	0.00
*B.cer*	5.41	71.93	1.63	17.75	0.59	2.70	0.00
*B.lic*	1.50	0.40	84.03	0.95	4.39	8.73	0.00
*B.meg*	3.04	35.64	3.22	38.41	8.86	10.82	0.01
*B.sph*	2.93	9.95	1.92	6.44	77.13	1.62	0.01
*B.sub*	4.20	0.61	4.64	1.69	1.05	87.81	0.01
*Br.lat*	0.01	0.03	0.00	1.13	7.28	0.11	91.43

*B.am B. amyloliquefaciens*, *B.ce B. cereus*, *Br.la Br. laterosporus*, *B.li B. licheniformis*, *B.me B. megaterium*, *B.sp B. sphaericus*, *B.su B. subtilis*

The findings in Table 4 can be summarized in three points:

1. *Br. laterosporus* did not match other species, which is not surprising because these bacteria are from a different genus.
2. Some species, including *B. cereus* and *B. megaterium*, are sometimes misclassified since they are phylogenetically related [3].
3. *B. subtilis* is sometimes misclassified with *B. licheniformis* and *B. amyloliquefaciens*.

Furthermore, heat maps of the confusion matrices were generated in order to visualize the classification of *Bacillus* strains. ESM Fig. S5A, B shows the heat maps generated for the seven species and 33 strains, respectively. Comparing the two heat maps that were generated from MALDI-TOF-MS (ESM Fig. S4A) and LC-MS (ESM Fig. S5A) when seven classes (species) are used, it can be seen that both techniques were robust at the species level, and both techniques showed that *B. megaterium* can be misclassified with *B. cereus*. Moreover, when 33 strains were compared, it can be seen that all the strains from *Br. laterosporus* showed the highest prediction accuracies. In addition, *B. subtilis* B0044 and *B. subtilis* B0098 overlapped and gave mixed classification results in both heat maps. These observations from LC-MS confirm that MALDI-TOF-MS is indeed a very useful and robust analytical technique which generates classifications similar to LC-MS.

LC-MS has relatively high resolution and sensitivity, and it also allows quantitative analysis to be performed. ESM Fig. S6 shows the relative levels of examples of the most significant lipids (based on the PCA loading plot) in the seven species classified in this study. ESM Table S4 shows a list of the putative assignment of the significant lipids. Again, based on the levels of these lipids, *Br. laterosporus* was observed to be significantly different in comparison with the other species, particularly based on fatty acid content (ESM Fig. S6A–D). Different lipids can be used to distinguish between species; for example, PE (14:1(9Z)/15:0) in ESM Fig. S6G could be used to distinguish *B. subtilis* from *B. amyloliquefaciens* and *B. licheniformis*. Significant lipids were also identified in the remaining 33 strains and are shown in ESM Fig. S7A–D. Table S5 in the ESM lists the putative assignment of significant lipids in the 33 strains. Looking back at ESM Fig. S7A, it can be noted that the existence of an unknown lipid is significantly higher in all the strains from *Br. laterosporus* compared to the remaining strains from *Bacillus*. Moreover, ESM Fig. S7B–D confirms that *B. subtilis* B0044 and *B. subtilis* B0098 are highly similar, and this is most likely due to producing similar amounts of lipids.

### Comparison of two analytical techniques

The objective of this step was to compare the patterns of *Bacillus* and *Brevibacillus* bacteria based on lipid extracts to those based on protein analyses, which have already been carried out previously using MALDI-TOF-MS [4]. In order to assess the similarities in the patterns that were generated from the two analytical techniques used for analysing lipids and proteins from *Bacillus* and *Brevibacillus* samples, three datasets were compared: MALDI-TOF-MS and LC-MS were used for the analysis of lipids and MALDI-TOF-MS for protein analysis. This led to the use of the Procrustean test. Table 5 shows the similarity between data obtained from the DFA plots for the seven species (highlighted in bold) and the 33 strains (in normal font). Table 5 highlights the following observations:

**Table 5:** Similarity between three different datasets for species and strain levels using Procrustes distance

	MALDI-MS lipid	MALDI-MS protein	LC-MS lipid
MALDI-MS lipid	–		
MALDI-MS protein	**0.1006** (***p*** **< 0.001**)	–	
	0.3443 (*p* < 0.001)		
LC-MS lipid	**0.0699** (***p*** **< 0.001**)	**0.1081** (***p*** **< 0.001**)	–
	0.3262 (*p* < 0.001)	0.4717 (*p* < 0.001)	

Values highlighted in bold correspond to seven classes (seven species) and those in normal font correspond to 33 classes (33 strains)

1. MALDI-TOF-MS lipid profiles and LC-MS lipid profiles had the highest similarity level, with a Procrustes distance of 0.0699 and a *p* value of <0.001 (i.e. not a single case where the permuted data obtained a lower Procrustes distance than that of the data without permutation). These findings were encouraging because this indicated bacteria were successfully classified using MALDI-TOF-MS analysis of lipids.
2. MALDI-TOF-MS protein profiles and both lipid-based experiments (MALDI-TOF-MS and LC-MS) were significantly similar, with Procrustes errors of 0.1006 and 0.1081 (*p* < 0.001), respectively. However, the errors are higher compared to that highlighted in point (1), which was expected as different compounds were compared (i.e. lipids and proteins), and as a result, this observation supports the validity of our work.
3. Data based on the 33 strains generated higher Procrustes errors compared to data on the seven species, and this is expected because of the larger number of strains compared to the number of species, hence the more complex data and the high similarity within a bacterial species. Nevertheless, the *p* values were still very significant (*p* < 0.001).

## Conclusion

MALDI-TOF-MS is an important technique in analysing biomolecular compounds and has been proven to be useful for discriminating between different microorganisms, and its use in bacterial profiling is common in clinical microbiology testing laboratories [59–61]. Our study involved the use of two analytical techniques, MALDI-TOF-MS and LC-MS, to analyse 33 strains from seven bacterial species belonging to the *Bacillus* (*n* = 6 species) and *Brevibacillus* (*n* = 1) genera. The spectral information generated using MALDI-TOF-MS on lipids extracted from the 33 strains and seven species was highly informative and was useful in discriminating between the bacteria at the subspecies level. In order to validate these findings, LC-MS data were used to evaluate and confirm the results obtained from the simple and rapid MALDI-TOF-MS analysis for bacterial classification. The results obtained from the two analytical techniques based on the seven bacterial species showed that these data were highly similar, which was supported by the use of Procrustes distance analysis. The calculated Procrustes distance was 0.0699 for the two datasets, indicating very high similarity between the MALDI-TOF-MS and LC-MS data. Finally, MALDI-TOF-MS data based on analysis of extracted lipids and previous analysis of proteins from intact bacteria of the same species were also very similar (Procrustes distance was 0.1006). These findings suggest that MALDI-TOF-MS can be used reliably as a powerful routine clinical tool for the robust classification and reliable identification of bacteria based on lipids or proteins.

## Electronic supplementary material

Below is the link to the electronic supplementary material.

ESM 1 (PDF 1325 kb)

ESM 2 (XLSX 40 kb)
