# Supplementary material for: Classification of Bacillus and Brevibacillus species using rapid analysis of lipids by mass spectrometry
Source: Anal Bioanal Chem. 2016 Sep 7;408(27):7865–78. doi: 10.1007/s00216-016-9890-4 (PMC5061856; doi:10.1007/s00216-016-9890-4)
Supplement: Supplementary file 1 — (PDF 1325 kb) [file 216_2016_9890_MOESM1_ESM.pdf]

Analytical and Bioanalytical Chemistry

Electronic Supplementary Material

Classification of Bacillus and Brevibacillus species using rapid analysis of lipids by mass spectrometry

Najla AlMasoud, Yun Xu, Drupad K Trivedi, Simona Salivo, Tom Abban, Nicholas J W Rattray, Ewa Szula, Haitham AlRabiah, Ali Sayqal, Royston Goodacre

216_2016_9890_MOESM2_ESM.xlsx

Figures & Tables

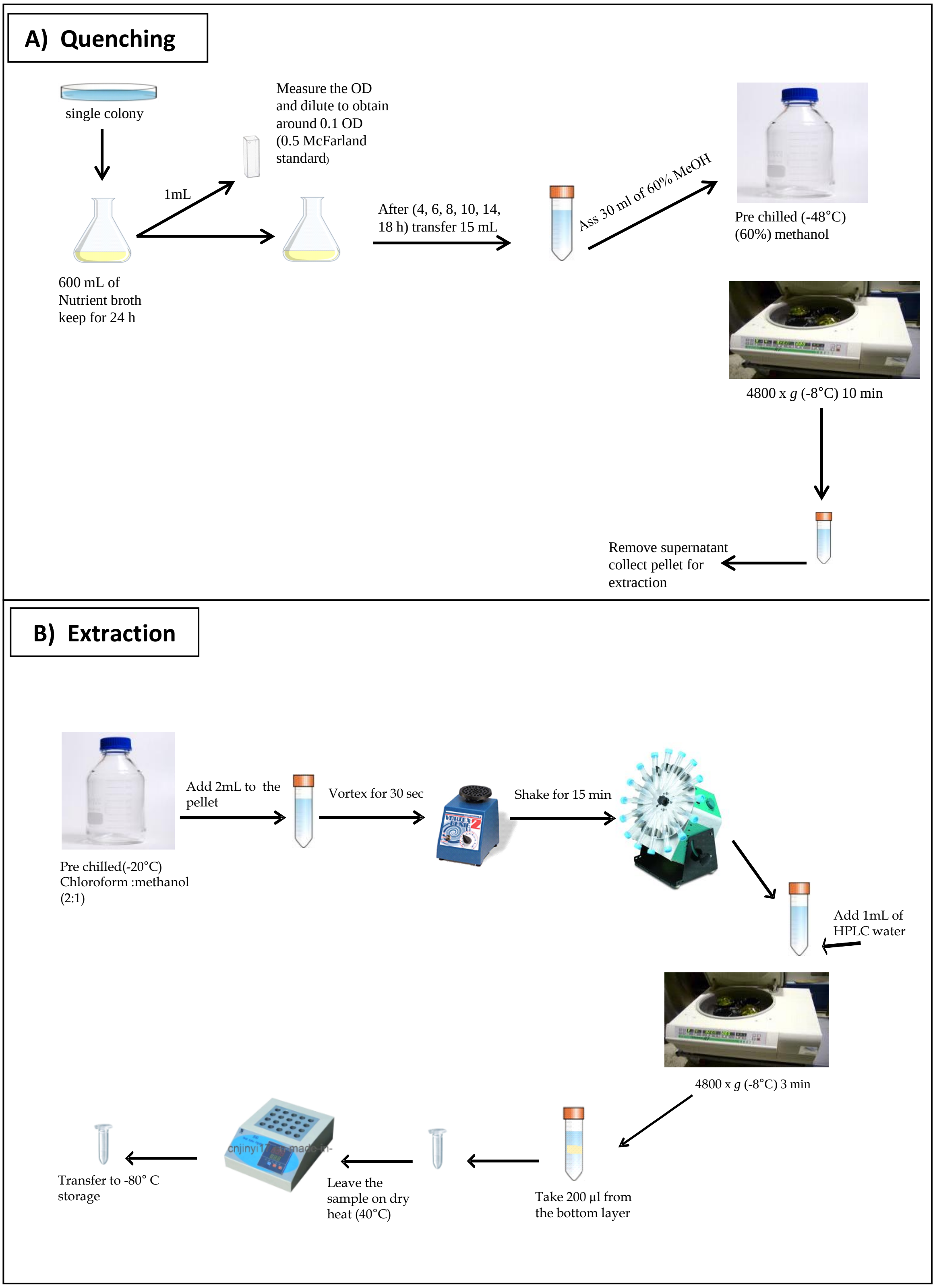

Fig. S1 (A) Schematic representing sample quenching using methanol (-48°C). (B) Schematic representing extraction of each sample for UHPLC-MS and MALDI-MS analysis using chloroform:methanol (2:1)

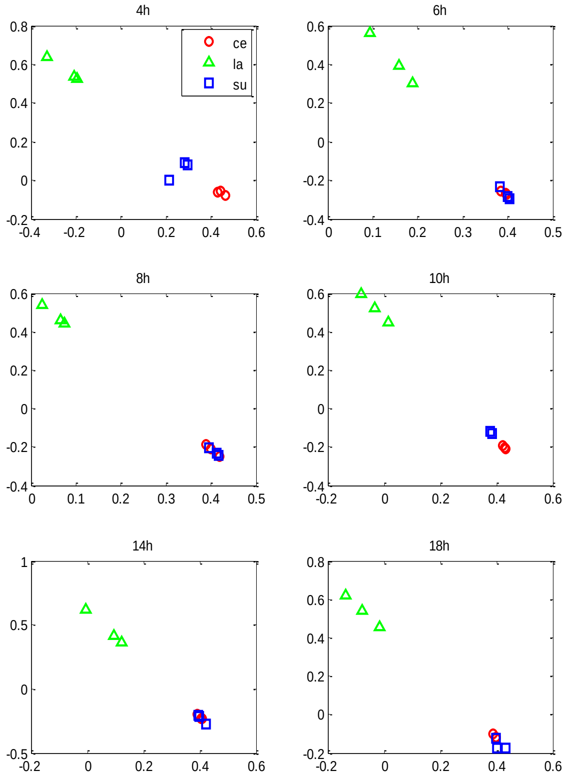

Fig. S2 UHPLC-MS Parallel factor analysis (PARAFAC2) at 4, 6, 8, 10, 14 and 18 h for three different species; where *B. cereus*= ce, *B. subtilis*= su & *Br. laterosporus*= la

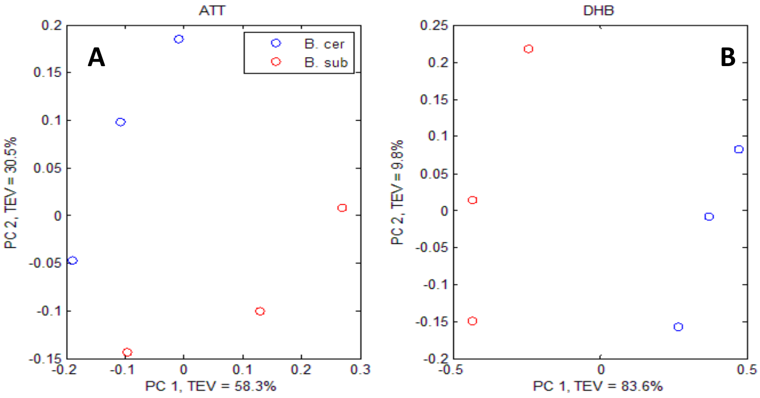

Fig. S3 PCA scores plots for two different *Bacillus* species: *B. cereus* and *B. subtilis* using two different matrices: (A) ATT and (B) DHB

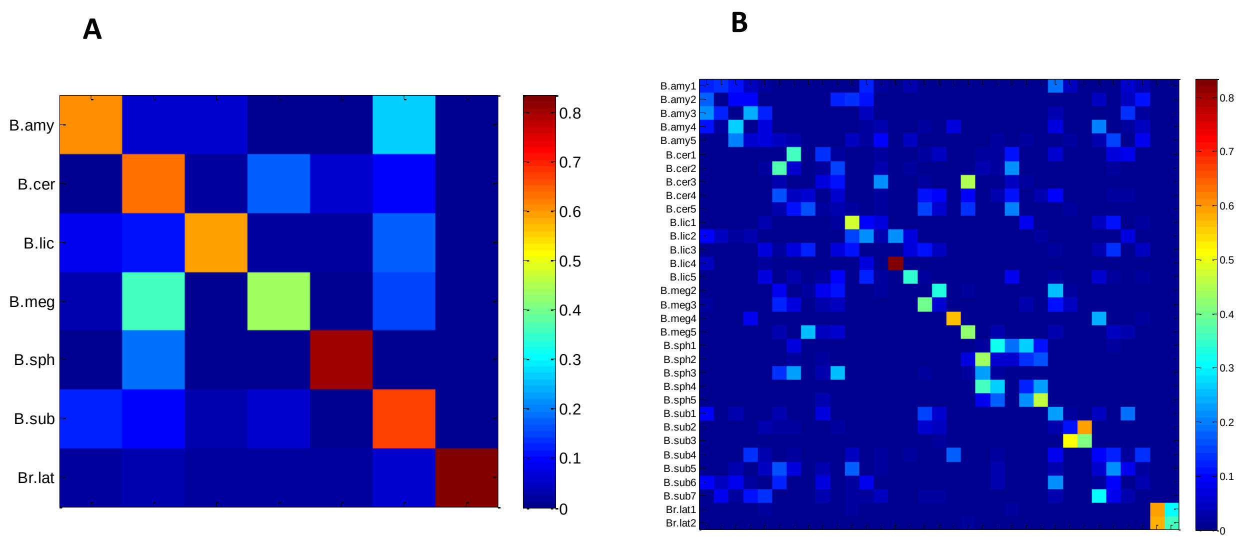

Fig. S4 Heat maps of the confusion matrices from: (A) 7 species and (B) 33 strains from *Bacillus* generated from PLS-DA on the MALDI-TOF-MS data

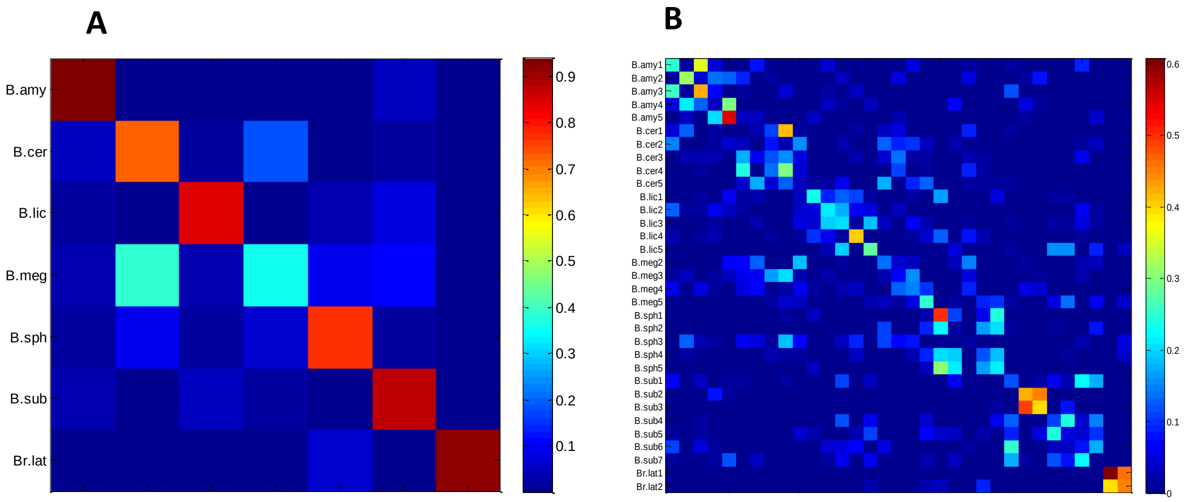

Fig. S5 Heat maps of the confusion matrices from: (A) 7 species and (B) 33 strains from *Bacillus* were generated from PLS-DA on the LC-MS data

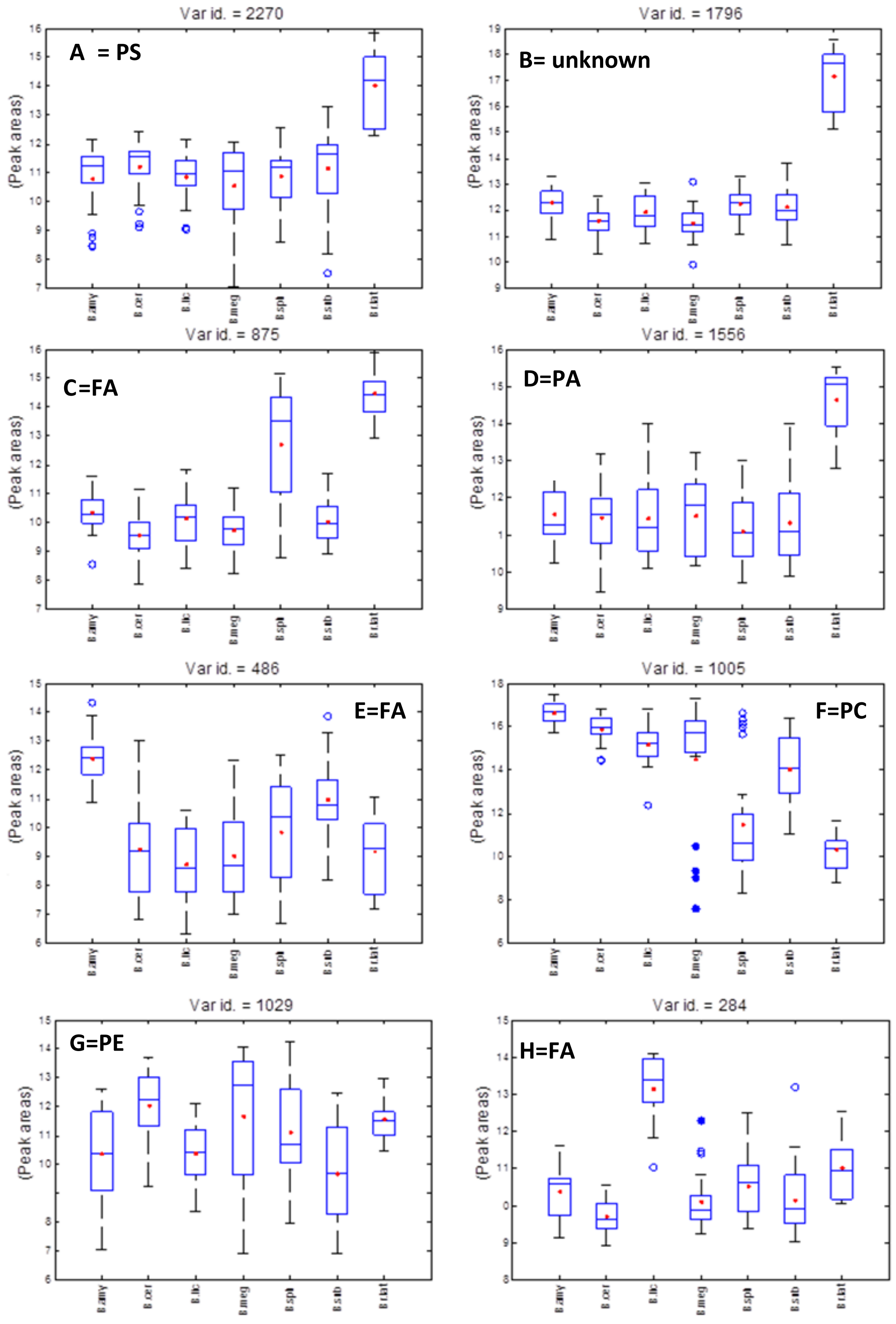

Fig. S6 Box-whisker plots for the seven species from *Bacillus* representing the relative concentration levels of different lipids (A-H). Each box plot indicates a different type of lipid; for more details see attached Excel sheet (Lipids-SI-TabsS3-S5.xls). X-axis coding: *B. amy: B. amyloliquefaciens, B. cer: B. cereus, Br. lat: Br. laterosporus, B. lic: B. licheniformis, B. meg: B. megaterium, B. sph: B. sphaericus* and *B. sub: B. subtilis.* For more information see Table S4 (Lipids-SI-TabsS3-S5.xls). Phosphatidylcholine (PC), phosphatidylethanolamine (PE), phosphatidic acid (PA) and fatty acid (FA)

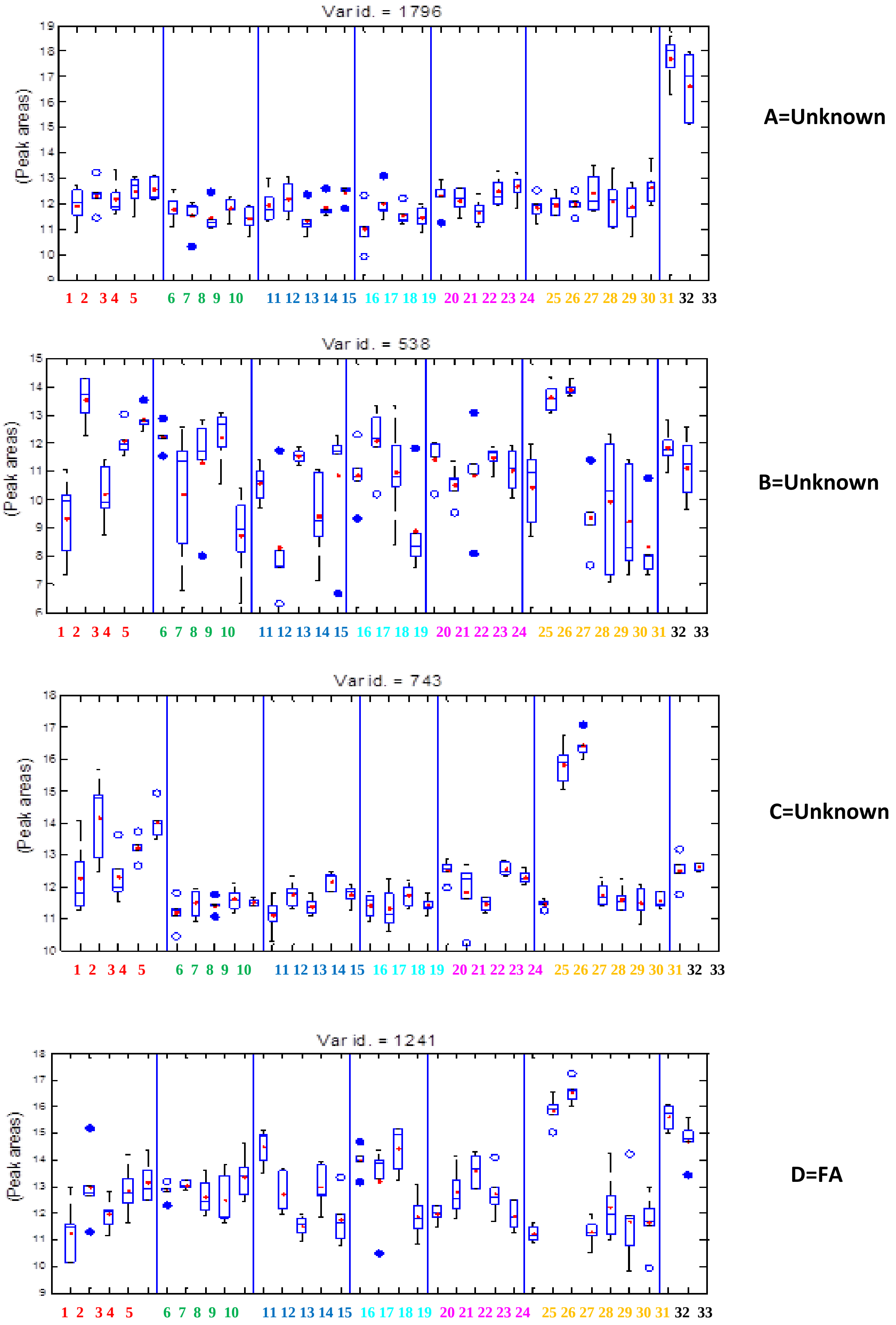

Fig. S7 Box-whisker plots (A-D) for the 33 strains representing the concentration levels of different lipids. Each box plot indicates a different type of lipids; for more details see Table S5. Each colour represents different strains as indicated in Table 1 on the manuscript. FA, fatty acids

Table S1 Prediction accuracies of the 33 *Bacillus* strains from MALDI-TOF-MS data using PLS-DA

	B.amy1	B.amy2	B.amy3	B.amy4	B.amy5	B.cer1	B.cer2	B.cer3	B.cer4	B.cer5	B.lic1	B.lic2	B.lic3	B.lic4	B.lic5	B.meg2	B.meg3	B.meg4	B.meg5	B.sph1	B.sph2	B.sph3	B.sph4	B.sph5	B.sub1	B.sub2	B.sub3	B.sub4	B.sub5	B.sub6	B.sub7	Br.lat1	Br.lat2
B.amy1	12.59%	14.04%	11.68%	4.92%	1.70%	0.02%	0.00%	0.13%	0.20%	0.37%	0.00%	12.21%	1.74%	0.11%	3.44%	0.20%	1.30%	0.00%	0.00%	0.00%	0.02%	0.22%	1.01%	0.00%	18.96%	4.39%	0.00%	0.75%	0.22%	6.22%	3.42%	0.00%	0.00%
B.amy2	17.43%	0.00%	9.30%	9.14%	1.30%	0.04%	0.65%	0.07%	0.00%	11.74%	14.09%	10.64%	0.00%	0.63%	0.00%	0.25%	0.09%	0.02%	0.90%	0.00%	0.29%	0.00%	0.20%	0.00%	0.04%	0.72%	1.28%	5.20%	0.00%	4.68%	11.18%	0.00%	0.00%
B.amy3	21.59%	12.47%	0.04%	24.63%	12.32%	1.02%	0.02%	0.00%	0.29%	0.00%	0.09%	5.13%	0.18%	0.04%	0.82%	0.00%	0.02%	0.00%	0.11%	0.00%	0.51%	0.60%	0.16%	0.04%	3.33%	0.00%	0.20%	0.00%	1.22%	13.14%	1.89%	0.00%	0.00%
B.amy4	11.40%	2.35%	26.13%	0.00%	6.66%	0.00%	0.70%	0.07%	0.07%	0.46%	0.15%	2.39%	2.79%	0.09%	0.28%	1.31%	0.04%	7.68%	0.24%	0.02%	0.02%	1.17%	0.04%	0.04%	7.77%	0.02%	0.20%	20.76%	0.00%	1.96%	5.00%	0.02%	0.04%
B.amy5	0.27%	0.65%	19.96%	5.26%	7.74%	4.68%	3.33%	0.00%	0.76%	0.43%	5.15%	2.01%	9.62%	0.00%	3.96%	0.38%	0.54%	0.00%	0.60%	0.00%	1.79%	0.60%	1.41%	0.22%	0.47%	2.44%	0.00%	3.80%	15.19%	0.07%	8.55%	0.00%	0.00%
B.cer1	0.00%	0.00%	0.27%	0.00%	0.02%	0.09%	35.88%	0.79%	13.67%	2.57%	0.00%	0.00%	1.96%	0.00%	0.32%	1.78%	5.07%	0.00%	0.83%	1.85%	2.01%	11.05%	0.18%	0.00%	5.68%	0.14%	0.00%	0.05%	7.31%	8.30%	0.05%	0.00%	0.00%
B.cer2	0.02%	0.00%	0.00%	0.00%	1.55%	36.76%	5.64%	0.00%	2.52%	14.48%	0.00%	0.00%	3.52%	0.00%	1.57%	0.20%	0.97%	0.00%	0.77%	3.50%	2.01%	21.05%	0.20%	0.31%	0.66%	0.58%	0.00%	0.49%	2.55%	0.51%	0.00%	0.00%	0.00%
B.cer3	0.00%	0.00%	0.00%	0.00%	0.00%	2.73%	0.89%	1.10%	6.99%	10.80%	0.00%	0.00%	21.96%	0.00%	0.00%	1.38%	0.11%	0.34%	45.55%	0.00%	0.91%	4.51%	1.31%	0.00%	0.00%	0.00%	0.00%	0.53%	0.32%	0.08%	0.19%	0.00%	0.17%
B.cer4	0.00%	0.00%	0.28%	0.00%	1.20%	16.56%	3.98%	6.20%	0.00%	6.14%	0.04%	0.00%	1.62%	0.00%	0.20%	10.83%	10.22%	0.02%	10.00%	0.07%	3.71%	10.62%	0.87%	3.23%	9.17%	0.48%	0.00%	0.13%	1.92%	1.97%	0.39%	0.00%	0.00%
B.cer5	0.11%	0.05%	0.00%	0.16%	0.02%	2.62%	10.89%	16.37%	2.03%	3.86%	2.41%	0.05%	1.56%	0.00%	0.79%	15.36%	5.82%	0.52%	13.37%	0.18%	0.05%	20.28%	0.38%	0.02%	0.09%	1.35%	0.02%	0.18%	0.00%	0.77%	0.56%	0.00%	0.00%
B.lic1	0.00%	0.16%	0.83%	0.20%	2.82%	0.07%	0.13%	0.00%	0.07%	1.45%	47.61%	10.20%	7.32%	0.00%	0.00%	0.40%	0.31%	0.09%	0.09%	0.11%	0.20%	0.65%	8.01%	1.07%	0.00%	0.02%	0.04%	4.34%	11.43%	0.74%	1.48%	0.02%	0.00%
B.lic2	9.42%	4.90%	2.00%	2.68%	1.26%	0.00%	0.00%	0.00%	0.09%	0.00%	15.10%	21.45%	3.09%	21.52%	7.51%	0.00%	0.00%	0.00%	0.00%	0.00%	0.00%	0.26%	0.00%	1.31%	0.04%	0.00%	0.00%	0.39%	0.07%	7.51%	1.28%	0.00%	0.00%
B.lic3	0.79%	0.24%	0.07%	0.77%	7.02%	1.34%	6.93%	12.28%	0.31%	6.84%	11.45%	1.29%	1.10%	0.00%	6.80%	10.57%	4.78%	0.02%	0.55%	0.07%	0.24%	0.46%	0.90%	0.83%	1.43%	1.12%	0.07%	3.60%	13.53%	0.18%	4.28%	0.04%	0.00%
B.lic4	4.97%	0.57%	0.07%	0.02%	1.86%	0.00%	0.00%	0.00%	0.09%	0.00%	1.24%	7.21%	0.00%	83.40%	0.00%	0.02%	0.00%	0.00%	0.00%	0.04%	0.00%	0.00%	0.00%	0.00%	0.00%	0.00%	0.00%	0.02%	0.00%	0.35%	0.00%	0.00%	0.00%
B.lic5	1.17%	0.00%	0.98%	0.04%	6.96%	1.13%	2.92%	0.17%	1.70%	9.20%	0.43%	13.03%	1.96%	0.02%	34.08%	2.38%	0.36%	0.09%	0.51%	0.09%	0.13%	9.11%	0.38%	0.02%	1.40%	0.30%	0.38%	6.51%	2.06%	0.32%	2.00%	0.04%	0.00%
B.meg2	0.55%	0.13%	0.20%	0.04%	0.18%	7.96%	0.51%	1.73%	8.05%	11.64%	0.00%	0.04%	1.55%	0.00%	0.38%	0.29%	33.13%	0.04%	2.02%	0.00%	0.00%	1.09%	1.13%	0.11%	25.66%	2.59%	0.16%	0.16%	0.00%	0.47%	0.02%	0.00%	0.04%
B.meg3	1.91%	0.46%	0.09%	0.00%	0.37%	11.93%	6.74%	0.00%	3.30%	4.87%	0.65%	0.00%	1.09%	0.00%	0.39%	39.81%	6.37%	0.00%	0.09%	0.39%	0.00%	0.17%	3.87%	0.89%	11.17%	3.98%	0.04%	0.15%	0.67%	0.24%	0.00%	0.15%	0.09%
B.meg4	0.48%	0.22%	0.33%	8.94%	0.18%	0.31%	0.24%	0.13%	0.18%	0.51%	0.09%	0.62%	0.33%	0.00%	0.07%	0.11%	0.84%	57.20%	0.22%	0.18%	0.33%	0.22%	0.18%	0.07%	0.11%	0.15%	0.00%	23.98%	1.21%	0.00%	2.47%	0.00%	0.00%
B.meg5	0.04%	0.00%	0.11%	0.00%	0.20%	2.95%	0.42%	25.21%	4.66%	5.51%	0.00%	0.00%	0.09%	0.00%	0.11%	1.18%	0.80%	0.60%	42.39%	0.00%	2.88%	0.29%	0.09%	0.22%	2.86%	0.07%	0.00%	0.54%	4.98%	3.48%	0.18%	0.00%	0.00%
B.sph1	0.00%	0.00%	0.00%	0.00%	0.00%	0.86%	7.42%	0.00%	0.00%	0.09%	0.00%	0.00%	0.00%	0.00%	0.00%	0.00%	0.02%	0.00%	0.00%	1.65%	32.06%	18.74%	26.20%	11.18%	0.00%	0.02%	0.00%	0.02%	1.56%	0.00%	0.00%	0.00%	0.04%
B.sph2	0.00%	0.00%	0.00%	0.16%	0.00%	0.78%	2.56%	0.62%	1.43%	0.09%	0.04%	0.00%	0.00%	0.00%	0.00%	0.00%	0.00%	0.00%	7.40%	44.07%	5.64%	5.26%	13.97%	16.24%	0.00%	0.00%	0.00%	0.02%	0.76%	0.80%	0.02%	0.00%	0.00%
B.sph3	0.00%	0.00%	0.11%	0.00%	0.20%	13.38%	23.09%	0.78%	3.68%	25.62%	0.63%	0.04%	0.00%	0.00%	0.26%	1.70%	0.04%	0.15%	2.00%	22.51%	1.61%	1.13%	0.22%	1.13%	0.00%	0.00%	0.00%	0.00%	0.52%	0.87%	0.13%	0.00%	0.04%
B.sph4	0.09%	0.00%	0.04%	0.00%	0.02%	0.00%	0.00%	0.00%	0.56%	1.14%	0.04%	0.06%	0.00%	0.00%	0.00%	0.67%	0.00%	0.00%	0.09%	35.25%	26.22%	0.00%	12.41%	23.10%	0.00%	0.00%	0.00%	0.00%	0.17%	0.02%	0.00%	0.00%	0.00%
B.sph5	0.00%	0.00%	0.00%	0.00%	0.00%	0.00%	0.09%	0.00%	3.50%	0.40%	0.54%	0.04%	0.07%	0.00%	0.00%	0.00%	0.00%	0.00%	0.40%	8.66%	17.83%	0.25%	21.95%	46.10%	0.00%	0.02%	0.00%	0.00%	0.00%	0.02%	0.00%	0.00%	0.00%
B.sub1	9.26%	0.00%	3.66%	0.34%	0.06%	3.75%	0.82%	0.00%	6.57%	0.17%	0.00%	0.19%	1.03%	0.00%	0.93%	14.92%	5.99%	0.00%	1.12%	0.00%	0.06%	0.06%	0.04%	0.00%	23.21%	2.15%	0.15%	4.63%	0.84%	19.32%	0.58%	0.00%	0.00%
B.sub2	0.80%	0.00%	0.00%	0.82%	2.91%	2.55%	2.40%	0.00%	0.28%	1.84%	0.00%	0.00%	1.25%	0.00%	0.17%	6.15%	5.14%	0.00%	0.00%	0.00%	0.00%	0.45%	0.00%	0.00%	3.52%	10.43%	59.89%	0.84%	0.04%	0.30%	0.09%	0.00%	0.00%
B.sub3	0.13%	0.02%	0.00%	0.00%	0.07%	0.00%	0.13%	0.00%	0.51%	0.70%	0.00%	0.00%	0.53%	0.00%	0.04%	1.23%	2.40%	0.00%	0.00%	0.00%	0.00%	0.04%	0.04%	0.00%	0.84%	51.50%	41.28%	0.33%	0.00%	0.00%	0.07%	0.00%	0.00%
B.sub4	1.28%	1.09%	0.22%	14.28%	3.71%	0.02%	1.84%	0.11%	0.15%	0.59%	4.88%	0.48%	2.13%	0.00%	1.80%	0.00%	0.00%	17.19%	0.82%	0.02%	2.04%	0.13%	0.00%	0.04%	8.03%	0.76%	0.00%	9.55%	12.78%	1.82%	14.11%	0.00%	0.00%
B.sub5	0.13%	0.00%	2.67%	0.04%	4.72%	16.31%	6.53%	0.00%	3.18%	0.02%	18.17%	0.20%	1.37%	0.00%	0.46%	0.00%	0.02%	0.00%	0.26%	0.79%	3.25%	0.02%	0.00%	0.15%	3.00%	0.11%	0.55%	6.36%	21.24%	8.59%	1.70%	0.00%	0.00%
B.sub6	9.84%	4.96%	9.09%	1.55%	0.62%	12.15%	0.29%	0.02%	7.71%	0.64%	2.55%	8.31%	0.00%	0.16%	0.18%	0.04%	0.00%	0.00%	0.62%	0.04%	2.88%	0.31%	0.00%	0.00%	21.21%	0.27%	0.04%	2.59%	9.22%	0.84%	3.72%	0.00%	0.00%
B.sub7	0.71%	7.86%	2.35%	11.46%	13.26%	0.00%	0.02%	0.69%	3.40%	0.47%	1.49%	1.68%	4.63%	0.00%	0.30%	1.36%	1.87%	1.03%	1.01%	0.00%	0.26%	0.15%	0.00%	0.09%	2.76%	0.11%	0.00%	30.90%	8.10%	3.25%	0.65%	0.00%	0.02%
Br.lat1	0.11%	0.00%	0.46%	0.00%	1.48%	0.04%	0.02%	0.00%	0.04%	0.17%	1.35%	1.11%	0.02%	0.00%	0.00%	0.00%	1.20%	0.00%	0.00%	0.00%	0.33%	2.17%	0.04%	0.00%	0.00%	0.67%	0.00%	0.02%	0.26%	0.61%	0.00%	59.10%	30.66%
Br.lat2	0.00%	0.00%	0.09%	0.00%	0.73%	0.09%	0.00%	0.00%	0.11%	0.02%	0.33%	0.07%	0.00%	0.00%	0.44%	0.18%	0.11%	0.00%	1.51%	0.02%	0.00%	0.86%	0.00%	0.33%	0.00%	0.16%	0.00%	0.09%	0.95%	0.24%	0.00%	57.40%	36.13%

The different colours represent the species level identification

Table S2 Prediction accuracies of the 33 *Bacillus* strains from LC-MS data using PLS-DA

	B.amy1	B.amy2	B.amy3	B.amy4	B.amy5	B.cer1	B.cer2	B.cer3	B.cer4	B.cer5	B.lic1	B.lic2	B.lic3	B.lic4	B.lic5	B.meg2	B.meg3	B.meg4	B.meg5	B.sph1	B.sph2	B.sph3	B.sph4	B.sph5	B.sub1	B.sub2	B.sub3	B.sub4	B.sub5	B.sub6	B.sub7	Br.lat1	Br.lat2
B.amy1	24.86%	1.30%	36.05%	5.64%	0.13%	0.42%	8.08%	0.04%	0.00%	0.00%	0.00%	3.97%	0.06%	0.77%	0.00%	0.00%	0.24%	5.07%	0.00%	0.00%	0.00%	1.02%	0.00%	0.00%	1.72%	0.00%	0.00%	0.00%	0.16%	9.06%	0.23%	0.00%	0.00%
B.amy2	1.40%	31.88%	3.91%	14.09%	12.60%	8.61%	0.00%	1.67%	0.15%	0.00%	0.00%	0.81%	3.63%	0.09%	0.00%	0.53%	4.96%	0.00%	0.24%	0.00%	0.00%	5.18%	0.33%	0.33%	0.00%	1.33%	8.01%	0.00%	0.00%	0.09%	0.00%	0.00%	0.00%
B.amy3	25.97%	1.77%	41.97%	7.06%	0.07%	1.04%	0.00%	0.03%	4.01%	0.08%	0.04%	1.12%	0.04%	2.89%	0.00%	0.00%	0.09%	0.68%	0.00%	0.00%	0.00%	0.46%	0.00%	0.00%	11.71%	0.00%	0.06%	0.04%	0.00%	0.44%	0.00%	0.00%	0.00%
B.amy4	5.32%	21.16%	12.60%	3.26%	29.96%	0.44%	0.58%	0.11%	0.00%	0.93%	0.19%	3.45%	1.67%	0.65%	2.93%	0.00%	0.00%	0.11%	0.00%	0.06%	6.79%	0.31%	0.31%	0.11%	0.06%	5.63%	1.74%	0.49%	0.00%	0.25%	0.45%	0.00%	0.00%
B.amy5	0.55%	3.62%	0.18%	20.04%	54.57%	3.19%	3.07%	0.00%	0.07%	0.00%	2.88%	0.19%	0.00%	0.00%	0.00%	0.07%	2.68%	0.29%	0.28%	0.00%	0.03%	0.00%	0.00%	0.14%	0.09%	1.40%	0.22%	0.92%	0.07%	0.00%	4.59%	0.00%	0.00%
B.cer1	5.06%	12.92%	0.90%	1.12%	0.84%	0.22%	2.33%	11.25%	41.13%	1.67%	0.86%	0.08%	0.04%	1.45%	0.06%	3.66%	5.16%	0.18%	0.11%	0.00%	0.23%	8.69%	0.06%	0.09%	1.34%	0.44%	0.00%	0.12%	0.00%	0.00%	0.00%	0.00%	0.00%
B.cer2	14.74%	0.07%	0.00%	1.64%	3.91%	2.96%	0.50%	8.30%	2.50%	16.00%	0.39%	0.24%	2.30%	0.56%	0.74%	13.18%	8.83%	10.04%	3.08%	0.00%	2.09%	0.85%	0.69%	0.00%	0.39%	0.47%	0.06%	0.00%	0.00%	1.07%	3.75%	0.00%	0.00%
B.cer3	0.24%	2.50%	2.72%	2.07%	0.17%	17.49%	6.08%	11.73%	15.80%	4.76%	0.37%	0.47%	0.20%	2.25%	0.00%	3.99%	13.52%	1.80%	1.54%	0.00%	0.06%	2.70%	0.98%	0.23%	0.04%	0.16%	0.15%	0.00%	0.09%	6.56%	0.00%	0.00%	0.00%
B.cer4	0.06%	0.35%	1.12%	0.00%	0.06%	24.22%	0.20%	14.06%	30.02%	4.57%	0.06%	0.00%	0.03%	0.00%	0.00%	0.04%	10.92%	0.48%	1.70%	0.00%	0.00%	8.55%	1.33%	0.71%	0.86%	0.44%	0.00%	0.00%	0.07%	0.15%	0.00%	0.00%	0.00%
B.cer5	0.05%	0.00%	0.63%	0.15%	0.00%	3.86%	17.76%	4.23%	13.02%	3.25%	1.22%	1.81%	5.70%	0.17%	0.00%	17.83%	0.79%	9.50%	12.43%	0.11%	0.22%	1.79%	1.34%	1.12%	1.34%	0.80%	0.00%	0.13%	0.25%	0.56%	0.00%	0.00%	0.00%
B.lic1	0.38%	0.03%	0.96%	0.09%	6.40%	1.72%	2.16%	0.09%	0.00%	3.51%	23.06%	8.75%	12.73%	11.36%	1.16%	0.03%	0.00%	1.03%	0.00%	17.04%	0.15%	0.00%	0.09%	5.64%	0.00%	0.00%	0.74%	1.43%	1.41%	0.00%	0.00%	0.00%	0.00%
B.lic2	12.99%	1.70%	1.27%	5.90%	3.33%	1.71%	0.14%	0.04%	0.00%	4.02%	5.66%	21.39%	17.49%	6.03%	5.26%	0.15%	3.41%	0.06%	0.04%	0.00%	0.04%	0.61%	0.00%	0.00%	0.10%	0.00%	0.00%	0.00%	0.58%	5.92%	1.74%	0.00%	0.00%
B.lic3	0.68%	0.13%	0.90%	0.99%	0.00%	0.00%	2.82%	0.10%	0.00%	5.02%	4.36%	19.56%	20.45%	1.36%	18.86%	3.05%	0.00%	7.58%	2.32%	0.00%	0.00%	1.22%	0.00%	0.00%	1.69%	0.00%	0.00%	1.85%	1.03%	5.02%	1.37%	0.00%	0.00%
B.lic4	2.52%	0.20%	1.88%	2.31%	0.00%	0.27%	0.09%	1.11%	0.00%	0.96%	10.35%	6.25%	1.54%	40.48%	0.06%	0.00%	0.26%	1.10%	4.49%	13.17%	0.41%	8.50%	1.99%	0.39%	0.00%	0.00%	0.00%	0.00%	0.00%	1.24%	0.00%	0.00%	0.00%
B.lic5	0.66%	0.00%	0.00%	0.14%	0.11%	0.00%	0.00%	0.00%	0.04%	0.00%	0.24%	2.07%	19.71%	0.06%	27.75%	0.58%	0.24%	0.07%	0.11%	0.10%	5.44%	0.21%	0.06%	0.00%	1.32%	0.03%	0.00%	16.00%	15.87%	0.06%	8.64%	0.00%	2.94%
B.meg2	0.00%	0.53%	0.00%	0.25%	7.51%	6.67%	13.26%	0.93%	0.55%	18.96%	0.04%	0.13%	1.76%	0.22%	0.46%	13.69%	4.62%	3.25%	0.07%	0.00%	3.49%	15.18%	0.00%	0.00%	0.00%	1.10%	0.40%	0.00%	0.32%	2.55%	3.19%	0.00%	0.00%
B.meg3	1.28%	3.64%	0.53%	1.55%	3.10%	6.10%	6.61%	16.50%	20.46%	2.48%	0.34%	2.61%	0.03%	0.30%	0.11%	2.04%	7.59%	15.23%	0.06%	0.19%	0.06%	5.11%	0.15%	0.00%	0.00%	0.06%	1.05%	0.00%	0.00%	2.63%	0.51%	0.00%	0.00%
B.meg4	6.45%	0.00%	4.76%	1.41%	0.06%	4.77%	7.31%	0.22%	1.31%	6.08%	1.85%	0.70%	2.77%	3.64%	0.16%	0.67%	13.19%	15.17%	9.83%	0.00%	0.00%	9.17%	0.60%	0.00%	0.00%	5.39%	4.42%	0.00%	0.05%	0.00%	0.00%	0.00%	0.00%
B.meg5	0.00%	0.00%	0.00%	0.00%	0.00%	0.48%	0.31%	1.86%	4.69%	3.31%	0.00%	0.06%	0.32%	0.65%	2.76%	2.98%	1.38%	5.27%	25.24%	0.26%	1.74%	0.09%	8.60%	10.10%	1.20%	0.00%	0.44%	5.57%	13.55%	0.60%	6.01%	0.00%	3.92%
B.sph1	0.00%	0.04%	0.82%	0.20%	0.00%	0.00%	0.14%	0.00%	1.41%	0.00%	3.02%	0.00%	0.00%	0.23%	0.00%	0.00%	0.00%	0.00%	2.30%	49.86%	10.99%	0.09%	6.47%	23.80%	0.00%	0.00%	0.00%	0.00%	0.00%	0.00%	0.00%	0.00%	0.00%
B.sph2	0.04%	0.00%	0.67%	0.94%	0.74%	0.00%	0.06%	0.06%	0.00%	0.26%	0.20%	0.00%	0.00%	0.39%	0.00%	11.05%	0.11%	0.15%	9.03%	22.28%	1.30%	0.33%	16.68%	20.85%	1.23%	0.29%	0.00%	1.29%	0.07%	0.09%	8.03%	0.00%	0.00%
B.sph3	0.11%	12.77%	2.22%	1.00%	0.00%	6.00%	4.89%	1.18%	18.12%	7.59%	0.00%	0.09%	2.91%	9.25%	0.00%	10.81%	7.01%	8.54%	0.09%	0.04%	0.37%	0.95%	0.15%	0.28%	0.07%	0.00%	0.50%	0.00%	0.00%	2.03%	0.00%	0.00%	4.90%
B.sph4	0.11%	0.99%	0.07%	0.86%	0.00%	0.06%	0.00%	2.02%	1.44%	1.22%	0.00%	0.10%	0.06%	2.90%	0.00%	0.06%	0.04%	2.83%	9.49%	20.64%	19.54%	0.04%	11.78%	18.28%	0.06%	0.00%	0.04%	0.00%	1.77%	0.25%	0.00%	0.49%	3.92%
B.sph5	0.00%	0.00%	0.94%	0.00%	0.00%	0.00%	0.07%	0.00%	0.24%	0.00%	3.09%	0.00%	0.06%	0.31%	0.00%	0.00%	0.04%	0.08%	3.34%	30.65%	21.14%	0.30%	16.30%	20.99%	0.13%	0.00%	0.39%	0.72%	0.00%	0.22%	0.80%	0.00%	0.00%
B.sub1	7.51%	0.11%	3.10%	0.08%	1.09%	1.23%	0.69%	0.03%	1.69%	0.50%	0.00%	0.47%	10.72%	0.11%	2.95%	0.00%	0.00%	0.12%	0.87%	0.11%	5.71%	0.27%	0.17%	0.43%	11.94%	0.04%	0.99%	6.00%	2.46%	22.24%	17.32%	0.00%	0.00%
B.sub2	0.03%	0.42%	0.00%	8.46%	1.81%	0.00%	0.59%	0.00%	0.09%	0.05%	0.00%	0.00%	0.00%	0.00%	0.00%	0.06%	0.05%	0.80%	0.00%	0.00%	0.03%	0.00%	0.00%	0.00%	0.00%	42.60%	45.11%	0.00%	0.00%	0.00%	0.00%	0.00%	0.00%
B.sub3	0.00%	0.50%	0.00%	0.59%	0.20%	0.00%	0.00%	0.00%	0.00%	0.00%	0.07%	0.00%	0.00%	0.00%	0.00%	0.40%	0.03%	0.46%	0.00%	0.00%	0.08%	0.00%	0.00%	1.07%	0.04%	49.06%	39.47%	0.04%	7.94%	0.00%	0.00%	0.00%	0.00%
B.sub4	0.00%	0.00%	1.60%	0.82%	0.28%	0.00%	0.00%	0.84%	0.00%	0.00%	0.15%	0.12%	12.04%	0.00%	5.95%	0.00%	0.00%	0.00%	4.38%	0.00%	0.10%	0.00%	0.00%	6.28%	12.61%	0.04%	0.00%	10.77%	24.38%	3.87%	14.77%	0.00%	0.00%
B.sub5	1.78%	0.00%	1.07%	0.13%	0.24%	0.04%	0.00%	0.08%	0.25%	3.89%	2.08%	0.44%	0.04%	1.98%	11.05%	0.15%	0.57%	0.10%	7.47%	4.20%	3.49%	0.00%	2.69%	0.24%	9.22%	0.07%	4.88%	24.18%	4.84%	3.90%	10.02%	0.00%	0.00%
B.sub6	11.17%	0.00%	5.36%	1.39%	0.00%	0.06%	0.19%	0.39%	0.00%	0.31%	0.00%	3.93%	5.24%	6.79%	0.05%	4.82%	0.71%	0.09%	0.00%	0.00%	0.00%	1.10%	0.00%	0.09%	25.12%	0.46%	0.00%	3.11%	3.31%	8.18%	17.80%	0.00%	0.00%
B.sub7	1.19%	0.00%	0.11%	1.37%	11.58%	0.00%	2.99%	0.11%	0.00%	0.46%	0.00%	1.37%	5.75%	0.00%	6.03%	0.00%	0.11%	0.17%	2.28%	0.00%	3.13%	0.34%	0.00%	0.63%	17.41%	1.73%	0.00%	11.89%	8.28%	22.18%	0.91%	0.00%	0.00%
Br.lat1	0.33%	0.00%	0.00%	0.00%	0.00%	0.00%	0.00%	0.00%	0.00%	0.00%	0.00%	0.00%	0.00%	0.00%	0.00%	0.00%	0.00%	0.00%	0.00%	0.00%	0.00%	0.00%	0.33%	0.00%	0.00%	0.00%	0.00%	0.00%	0.00%	0.00%	0.00%	60.78%	46.08%
Br.lat2	0.00%	0.00%	0.00%	0.00%	0.00%	0.00%	0.00%	0.00%	0.00%	0.00%	0.00%	0.00%	0.00%	0.00%	1.96%	0.00%	0.00%	0.00%	1.96%	2.94%	0.00%	0.00%	8.82%	0.00%	0.00%	0.00%	0.00%	0.00%	0.00%	0.00%	0.00%	39.22%	45.10%

The different colours represent the species level identification
